# An Approach for Magnetic Halloysite Nanocomposite with Selective Loading of Superparamagnetic Magnetite Nanoparticles in the Lumen

**DOI:** 10.1021/acs.inorgchem.0c01039

**Published:** 2020-08-12

**Authors:** Hady Hamza, Anna Maria Ferretti, Claudia Innocenti, Katarzyna Fidecka, Emanuela Licandro, Claudio Sangregorio, Daniela Maggioni

**Affiliations:** † Dipartimento di Chimica, Università degli Studi di Milano, Via Golgi 19, 20133 Milano, Italy; ‡ SCITEC−CNR, Sede Secondaria via G. Fantoli 16/15, 20138 Milano, Italy; § ICCOM-CNR, via Madonna del Piano 10, 50019 Sesto, Fiorentino, Italy; ∥ Consorzio INSTM, Via G. Giusti, 9, 50121 Firenze, Italy; ⊥ Dipartimento di Chimica, Università degli Studi di Firenze, via della Lastruccia 3, 50019 Sesto, Fiorentino, Italy

## Abstract

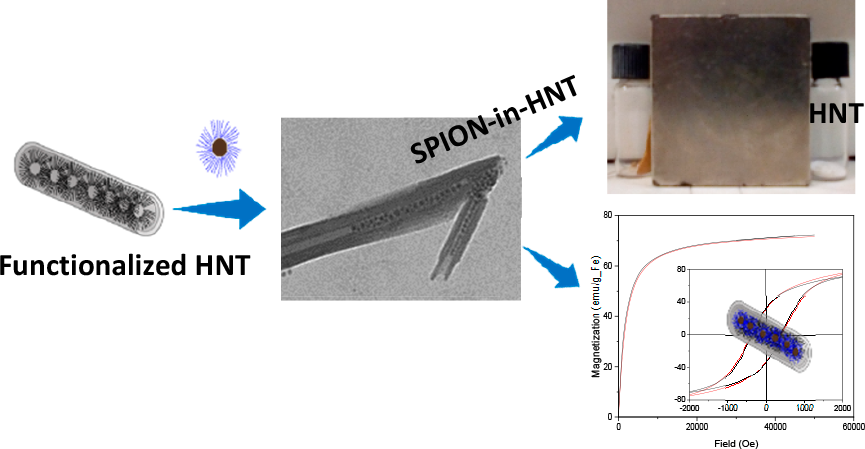

We present for the first time a method for the preparation of magnetic halloysite nanotubes (HNT) by loading of preformed superparamagnetic magnetite nanoparticles (SPION) of diameter size ∼6 nm with a hydrodynamic diameter of ∼10 nm into HNT. We found that the most effective route to reach this goal relies on the modification of the inner lumen of HNT by tetradecylphosphonic acid (TDP) to give HNT–TDP, followed by the loading with preformed oleic acid (OA)-stabilized SPION. Transmission electron microscopy evidenced the presence of highly crystalline magnetic nanoparticles only in the lumen, partially ordered in chainlike structures. Conversely, attempts to obtain the same result by exploiting either the positive charge of the HNT inner lumen employing SPIONs covered with negatively charged capping agents or the *in situ* synthesis of SPION by thermal decomposition were not effective. HNT–TDP were characterized by infrared spectroscopy (ATR-FTIR), thermogravimetric analysis (TGA), and ζ-potential, and all of the techniques confirmed the presence of TDP onto the HNT. Moreover, the inner localization of TDP was ascertained by the use of Nile Red, a molecule whose luminescence is very sensitive to the polarity of the environment. The free SPION@OA (as a colloidal suspension and as a powder) and SPION-in-HNT powder were magnetically characterized by measuring the ZFC-FC magnetization curves as well as the hysteresis cycles at 300 and 2.5 K, confirming that the super-paramagnetic behavior and the main magnetic properties of the free SPION were preserved once embedded in SPION-in-HNT.

## Abstract 2

SPION nanoparticles are selectively loaded into halloysite lumen, keeping their superparamagnetic character.

## 1 Introduction

**Figure 1:**
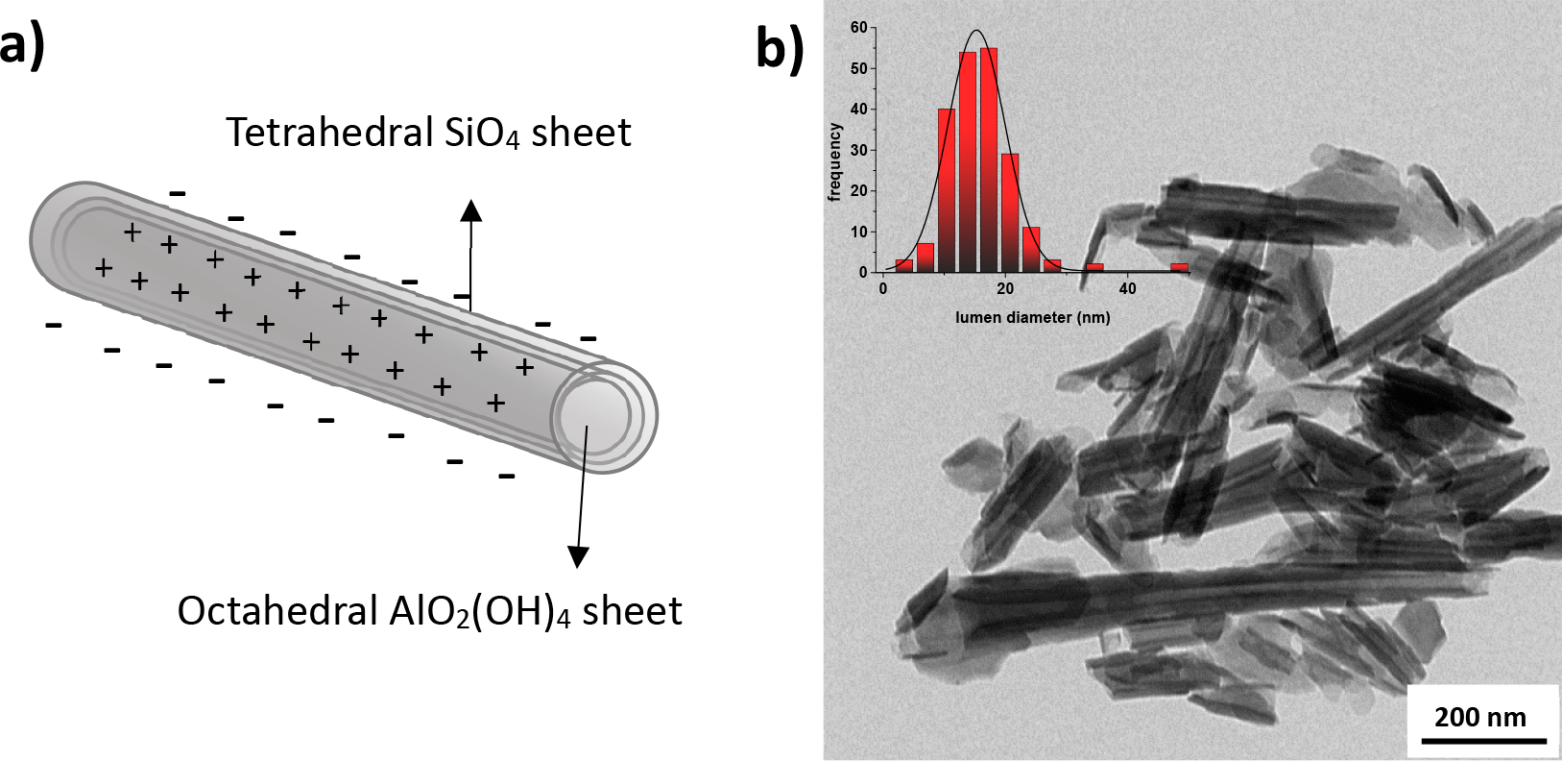
(a) Schematic representation of the structure of a halloysite with the indication of the molecular geometry around Al and Si centers specific for each layer and (b) TEM micrograph of a HNT sample (inset: HNT lumen diameter distribution).

Halloysite nanotubes (HNT) are unique natural nanomaterials composed by a double-layered aluminosilicate with a hollow tubular structure (Figure 1a) in the microrange and an inner diameter usually ranging between 15 and 50 nm (Figure 1b).

The chemical composition of the HNT inner and outer parts is different. Specifically, the inner surface is made by alumina with exposed aluminum hydroxide groups (Al–OH), while the outer one is made by silica [siloxane groups (Si–O–Si)].^1^ The structural formula of HNT is Al_2_(OH)_4_Si_2_O_5_·(H_2_O)_2_ when it is in its hydrated form.^2^ Due to the different chemical compositions of the outer and the inner parts, the HNT inner lumen is positively charged due to the protonation of exposed OH groups as well as the possible coordination vacancy at Al^3+^ sites.^3^ Instead, the outer part is negatively charged, due to the few OH groups only present in the structural defects of the siloxane layer and at the edges of the nanotubes.^4^ The potentiality for the industrial development of HNT-based derivatives is more than probable, due to the low price, ecofriendly properties, and biocompatibility of this material, as well as facile and selective functionalization of the HNT two layers with different functional groups.^5,6^

Thanks to these features, HNT have been already employed as possible drug delivery vectors^6−8^ for proteins and small drugs,^9,10^ since negatively charged drugs can be easily retained by the positive lumen, which could act as an inorganic nanocapsule for controlled release.^11,12^ Conversely, long oligonucleotide strands, despite the negative charge, cannot be loaded in the HNT inner lumen possibly due to the large size. Nevertheless, they can be delivered by immobilizing them on the outer surface through different strategies.^13−16^ As regards HNT morphology, HNT can be considered as an alternative to multiwalled carbon nanotubes for certain applications, with the advantages of being naturally available, durable, very cheap (HNT $4 per kg, while carbon nanotubes $500 per kg),^17^ and more biocompatible than carbon nanotubes. HNT have been also proposed for the delivery of other active chemicals such as anticorrosion agents and flame retardants, as well as nanoreactors for enzymatic biocatalysts and water remediation agents for heavy metal ions removal.^7,18,19^

Superparamagnetic iron oxide nanoparticles (SPION) made of magnetite (Fe_3_O_4_) have been studied in depth in the last decades especially for applications in the biomedical field.^20−25^ Indeed, their superparamagnetism is essential for safe use *in vivo*, since their total magnetization is null in the absence of an applied external magnetic field, thus preventing any aggregation event that could cause capillary occlusion. They possess many favorable features for both imaging and therapy: First, they are biocompatible and biodegradable,^26,27^ and they can be exploited as contrast agents in magnetic resonance (MRI), as drug delivery carriers, for various separating techniques, and as heat mediators for magnetic fluid hyperthermia (MFH) treatments, among others.^28^ SPION have also been extensively used for triggering the drug release from several different nanocomposites^29−32^ or nanoporous systems.^33^

Due to the biocompatibility of both HNT^3^ and SPION, many previous research studies focused on the preparation of HNT–SPION nanocomposites, but in the most cases the SPION were anchored on the outer surface of HNT^34−41^ or grown by coprecipitation in the HNT lumen.^42,43^ In this last case, the crystallinity, the size of the nanoparticles, and their magnetic properties were hardly controllable. To the best of our knowledge, preformed magnetic NPs have never been selectively loaded in the inner lumen of HNT so far.

In contrast, the loading of preformed SPION with shaped properties would be of interest for many applications, such as MRI or MFH. Moreover, the inner loading could give rise to a higher oxidative resistance of NPs compared to their anchoring on the HNT external surface. In fact, HNT could act as a protective barrier slowing down the molecular oxygen action, as recently previewed for carbon nanotubes filled with SPION.^44^

In the literature, there are many examples of *in situ* formed metallic, quantum dot, oxide NPs, selectively loaded onto or into HNT,^45^ while only very few examples report loading of preformed nanoparticles (NPs) in the HNT lumen. All of these deal with nonmagnetic NPs. Specifically, one concerned silver NPs of 2 nm diameter stabilized by citrate anions loaded by exploiting the opposite charge of the positive HNT lumen and negative NPs.^46^ The same procedure was recently exploited for the loading of palladium NPs.^47^ In another example, 2 nm carbon nanodots were loaded into the lumen by exploiting the vacuum pouring technique.^48^ In all of the other studies, the NPs were grown in the presence of HNT,^49^ but in many cases, the growing NPs interacted with the outer surface instead of the inner one. Only a few examples describing the selective inner growth have been reported to date: The first one showed the ability of Au NPs to selectively form in the HNT lumen;^50^ the same was achieved for Ru-based metallic clusters.^51^ The last one reported the possibility of growing iron oxide NPs made by a coprecipitation method.^43^

The goal of this work was to find a reliable and reproducible method to selectively fill the HNT lumen with preformed SPION possessing well-defined and possibly good magnetic properties. The final aim of this study was to obtain HNT–SPION as a “building block” for further developments of suitable nanocomposites. The nanocomposites based on SPION-in-HNT could be useful for different applications, such as (i) in biomedical field, as new theranostic agents for MRI, MFH, and controlled release of a drug by an external magnetic stimulus, (ii) in catalysis, with the double advantage to recover the system due to the magnetic NPs in the inner lumen leaving the external HNT surface available for further decoration with catalytic organometallic compounds or other types of nanoparticles, (iii) in water remediation, by exploiting a capturing agent or a photoreactive organometallic compound anchored on the external surface while maintaining the ability to magnetically recover the nanocomposite, and (iv) in tissue engineering for the development of 3D scaffolds able to align cells of an anisotropic growing tissue.

## 2 Results and Discussion

**Scheme 1:**
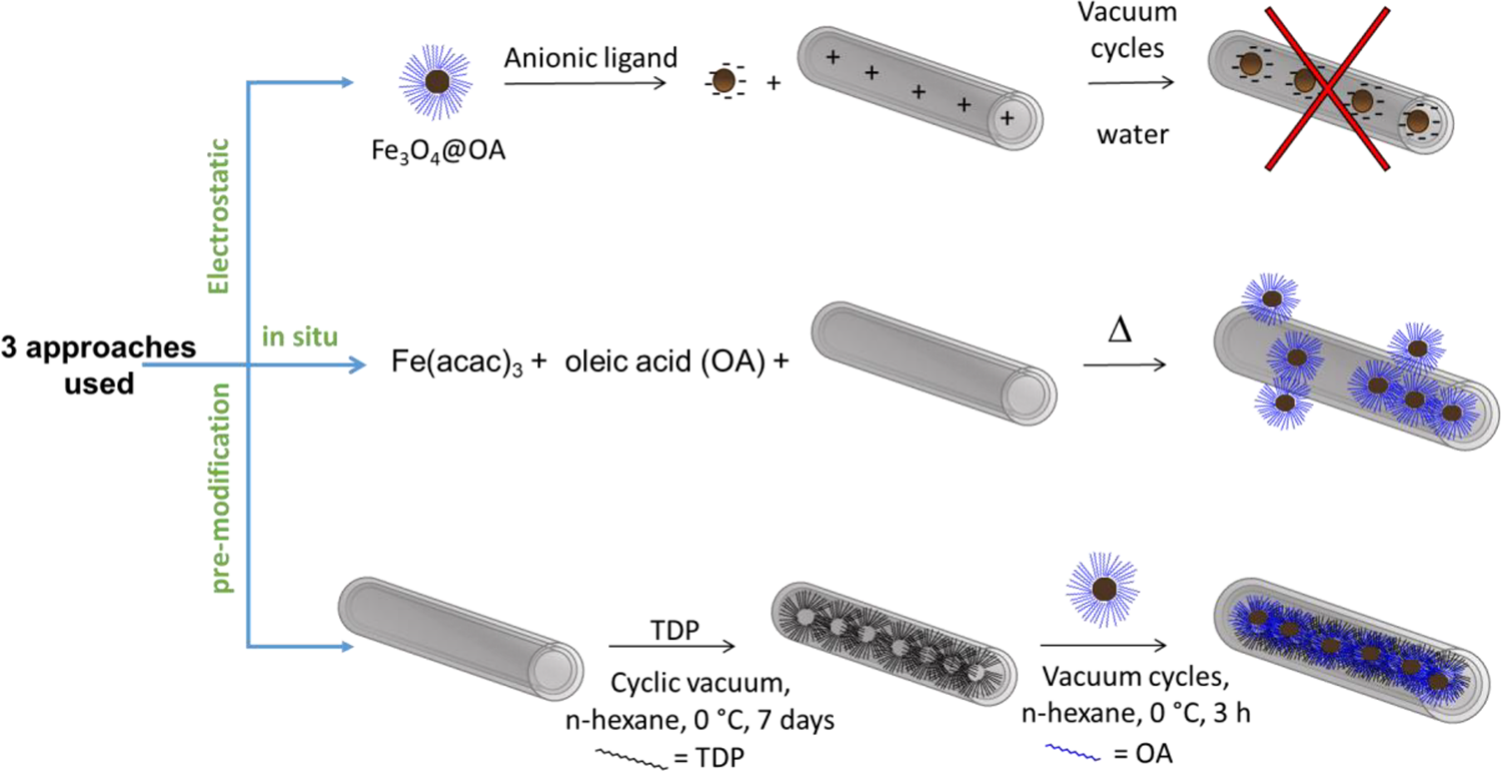
Schematic Depiction of the Three Distinct Approaches Followed in This Study to Obtain HNT–SPION Nanocomposite with the Selective Loading of SPION in the Inner Part

In order to fill the HNT with magnetic NPs, we investigated three distinct approaches (depicted in Scheme 1): electrostatic interaction, *in situ* formation of SPION, and premodification approach. All three strategies are described below, even though only the third one gave us the desired HNT–SPION adduct.

### 2.1 Attempts to Prepare SPION-in-HNT Using Water-Dispersible Negatively Charged SPION: The Electrostatic Approach

The first method tried to exploit the charge difference between the inner and the outer part of the HNT. Indeed, the inner layer of alumina remains positively charged up to pH 8.5, while the external silica layer is negatively charged for almost all the pH range above 1.5.^3^ This charge difference has been extensively exploited for the loading of small negatively charged molecules into HNT inner lumen and their forward sustain release at intended site.^52^ Hence, despite the overall negative charge of HNT at physiological pH, the inner alumina remains positively charged to a certain extent. As already mentioned in the Introduction, just a few examples were reported on the loading of preformed NPs in the HNT lumen.^46−48^ One of them was carried out using small negative Ag NPs.^46^ In that case, the prepared NPs were tiny (ca. 2.6 nm), and the loading procedure was extremely simple and involved the use of neither sonication nor vacuum cycles to induce the NPs to enter into the HNT lumen. Thus, it can be concluded that the diffusion by Brownian motion was effective enough to fill the HNT lumen with NPs.

We tried to follow the same approach but applying vacuum/N_2_ cycles in order to exploit the strong capillary pressures affecting the HNT lumen and taking into account that the HNT inner diameter measured by TEM was ∼15.3 ± 0.3 nm. Hence, we prepared negatively charged SPION of a suitably small size, ranging from 5.1 to 6.9 nm. This size was judged to be a good compromise between the need of employing NPs small enough to enter the lumen but, at the same time, big enough to maintain good magnetic properties. We adopted different approaches for the SPION synthesis (resumed in Table 1), passing from a simple coprecipitation method^53^ to thermal decomposition syntheses.^54,55^ Indeed, one of the main goals of this work was the development of a strategy to fill HNT with the best NPs in terms of magnetic properties. It is well-known that for SPION the best magnetic properties are achievable through thermal decomposition syntheses able to modulate size and crystallinity.^56^

**Table 1:** Mean Size (Diameter, nm) of Synthesized SPION as Measured by TEM and DLS in Water Suspensions^a^

					DLS/nm	
sample ID	ref	NPs synthesis method	NP stabilizer^b^	TEM/nm	hexane^c^	water
SPION1@DMSA	(53)	coprecipitation with NaOH	DMSA^58^	5.3 ± 1.0	13.1 ± 3.0	13.5 ± 2.4
SPION2@DMSA	(53)	coprecipitation without NaOH	DMSA^58^	d	10.6 ± 4.0	
SPION3@DMSA^e^	(54)	thermal decomposition using Fe(acac)_3_	DMSA^58^	5.1 ± 1.6	8.0 ± 1.9	
SPION4@GA	(55)	thermal decomposition using iron oleate	GA^22^	6.9 ± 1.0	10.7 ± 2.3	13.0 ± 2.5
SPION4@TMAOH	(55)	thermal decomposition using iron oleate	TMAOH^59^			10.0 ± 3.0
SPION4@PA	(55)	thermal decomposition using iron oleate	PA			13.0 ± 4.0
SPION3@OA^e^	(54)	thermal decomposition using Fe(acac)_3_	OA	6.1 ± 1.3	10.9 ± 3.0	

a The synthetic method used is indicated together with the relative reference.

b DMSA = dimercaptosuccinic acid; GA = gallic acid; TMAOH = tetramethylammonium hydroxide; PA = protocatechuic acid; OA = oleic acid.

c The DLS measurements were performed in *n*-hexane suspension on the as-prepared SPION@OA.

d TEM images were not acquired on this sample.

e The synthetic procedure for the preparation of SPION@OA was the same, but repeated twice, leading to slightly different mean size values.

In Table 1 we reported the data of the several essays we made with the electrostatic attraction approach. These implied a slight variation of the SPION dimension, but most of all, the variation of the surface capping ligands that in vain were changed, trying to encourage the entry of negatively charged SPION into the nanotube lumen avoiding early SPION aggregation. Despite all the efforts made, none of the attempted procedures was successful. All the experimental details, a related discussion and figures (Figures S1–S8) are reported in the Supporting Information.

We therefore concluded that the electrostatic attraction, useful for small-molecule loading in HNT lumen,^10,11,52^ as well as for tiny nonmagnetic NPs,^46−48^ was not effective in the case of these magnetic nanoparticles, regardless of the SPION size or coating. This could be due to the SPION mutual attraction in water that can be reinforced by additional attractive forces such as hydrogen bonds between different particles, especially when they are in the restricted space volume at the entrance of the halloysite lumen under conditions of reduced pressure.

### 2.2 Attempts of SPION-in-HNT Preparation Using Thermal Decomposition in the Presence of Pristine HNT

In this case we tried to carry out an *in situ* thermal decomposition synthesis of SPIONs in the presence of HNT as reported in the literature^57^ (Scheme 1). The chosen method for the synthesis of SPIONs was in principle able to lead to highly magnetized and monodispersed SPIONs into HNT. Thus, the iron precursor and the other reactants were made diffusing into the lumen of HNT reducing the pressure in the vessel for some minutes before starting the reflux, to ensure the diffusion into the lumen of the reaction mixture. HNT–SPION adduct was washed thoroughly using ethanol by centrifugation. The light brown powder recovered was magnetic, being attracted by a neodymium–iron–boron magnet and was dispersible in water. Unfortunately, TEM analysis of the obtained product showed that even if some SPION were localized inside the HNT (highlighted in Figure S2 by red arrows) then they were also found attached to the external surface, showing that the growth of NPs was not selectively confined to the HNT lumen. Moreover, SPION were not highly monodispersed, and owing to the effect of high temperature, the outer surface of HNT was partly etched.

### 2.3 SPION-in-HNT by Functionalization of the Inner Lumen of HNT with Tetradecylphosphonic Acid

The failed attempts obtained by pursuing the two previous paths prompted us to verify whether the SPION could be inserted in their native state, that is, with the apolar capping agent (oleate) still on the surface, by properly modifying the lumen polarity of HNT. From the literature, it is known that it is possible to selectively functionalize the lumen of HNT using a phosphonic acid, which preferentially reacts with alumina compared to silica.^60,61^ The inner surface of HNT was successfully modified with tetradecylphosphonic acid (TDP).^61^ To ascertain whether the reaction had taken place, we employed several analytical techniques. As a first qualitative assay, we used Nile Red dye. Indeed, this molecule is very sensitive to the environment in which it is dispersed, in that both the absorption and the emission are heavily perturbed.^62^ Nile Red is highly emissive in hydrophobic environments, such as the one present in the functionalized lumen with TDP, while it is completely quenched in water. After the loading of Nile Red (see the Experimental Section for details) the HNT–TDP sample turned purple; hence, they were thoroughly washed with water to remove all the possible externally interacting dye. The still wet purple HNT–TDP sample, observed under UV light irradiation, exhibited a red luminescence. On the contrary, the pristine HNT sample treated in the same way with Nile Red and washed with water turned purple as well, but it did not show any emission under the UV light irradiation, as, on the contrary, the HNT–TDP sample did. This behavior was ascribed to the different microenvironment in which the dye is located: In the case of HNT–TDP sample, the Nile Red is likely intercalated into the apolar long aliphatic chains, thus preserving Nile Red from the quenching provoked by its direct interaction with water (Figure S10).

**Figure 2:**
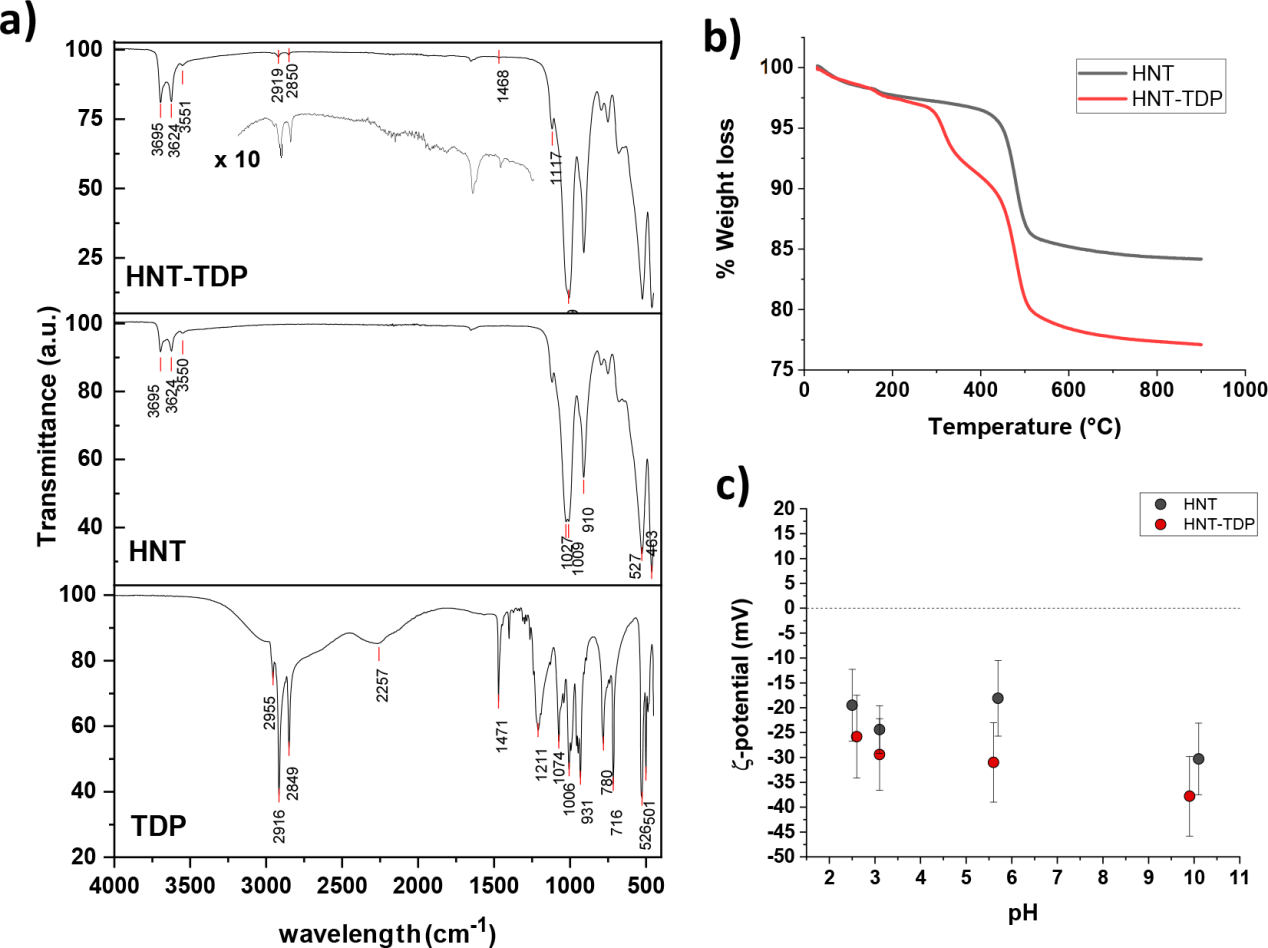
Characterization of the HNT–TDP (a) FTIR spectrum of HNT–TDP (top; the inset shows the magnified region of the spectrum containing signals of TDP) compared with the FTIR spectra of HNT (middle) and TDP (bottom); (b) thermogravimetric analysis (TGA) of pristine HNT and HNT–TDP derivative; (c) ζ-potential analyses of suspensions of HNT and HNT–TDP.

ATR-FTIR spectroscopy (Figure 2a) and thermogravimetric analysis (TGA, Figure 2b) confirmed the successful functionalization of halloysites. The ATR-FTIR spectrum of HNT–TDP (Figure 2a, top), as well as that of pristine HNT (Figure 2a, middle), showed the characteristic sharp bands related to Al–OH stretching of lumen and interlayer alumina peaking at 3692 and 3624 cm^–1^, respectively, and of the OH stretching of hydrogen-bonded water present in the interlayers (3551 cm^–1^).^60^ The spectrum of HNT–TDP showed the stretching bands of the aliphatic chain in the region between 3000 and 2800 cm^–1^ (2959, 2919, and 2851 cm^–1^), together with the deformation (scissoring) of CH_2_ groups (1468 cm^–1^), while the bands due to the C–P–O stretching in the region 1100–800 cm^–1^ overlapped with the very intense bands of HNT, hampering their visualization and attribution. Finally, the broad P–O–H band for the unbound TDP visible in the TDP spectrum (bottom) at ∼2300 cm^–1^ as well as the complete disappearing of the P=O band vibration at 1211 cm^–1^ suggest that TDP is bonded to the halloysites, and in particular to the alumina lumen layer, in a deprotonated form.^60,61^

TGA analysis of HNT (black trace of Figure 2b) showed a mass loss step (ca. 2.5%) corresponding to the loss of adsorbed water on surface (onset *T* = 45 °C) and into interlayer (onset *T* = 163 °C), and a second mass loss step (ca. 14.6%, onset *T* = 450 °C, inflection point at 480 °C) assigned to the dehydroxylation of structural AlOH groups of halloysites.^63^ Differently, the TGA profile of HNT–TDP (red trace, Figure 2b) showed an extra mass loss step (ca. 5.4%, onset *T* = 300 °C, inflection point at 320 °C) attributed to the degradation of TDP.

We carried out also the analysis of ζ-potential before and after the treatment with TDP, and the obtained values as a function of pH are shown in Figure 2c. The observation of a more negative value for HNT–TDP is not only a further confirmation of the interaction of the HNT with the TDP but also indirectly suggests the TDP localization is in the inner part of the nanotube. Indeed, if the phosphonic acid mostly interacts with the positively charged inner part, then it neutralizes a portion of these charges, thus it making slightly more negative the whole nanotube, as experimentally observed.

**Figure 3:**
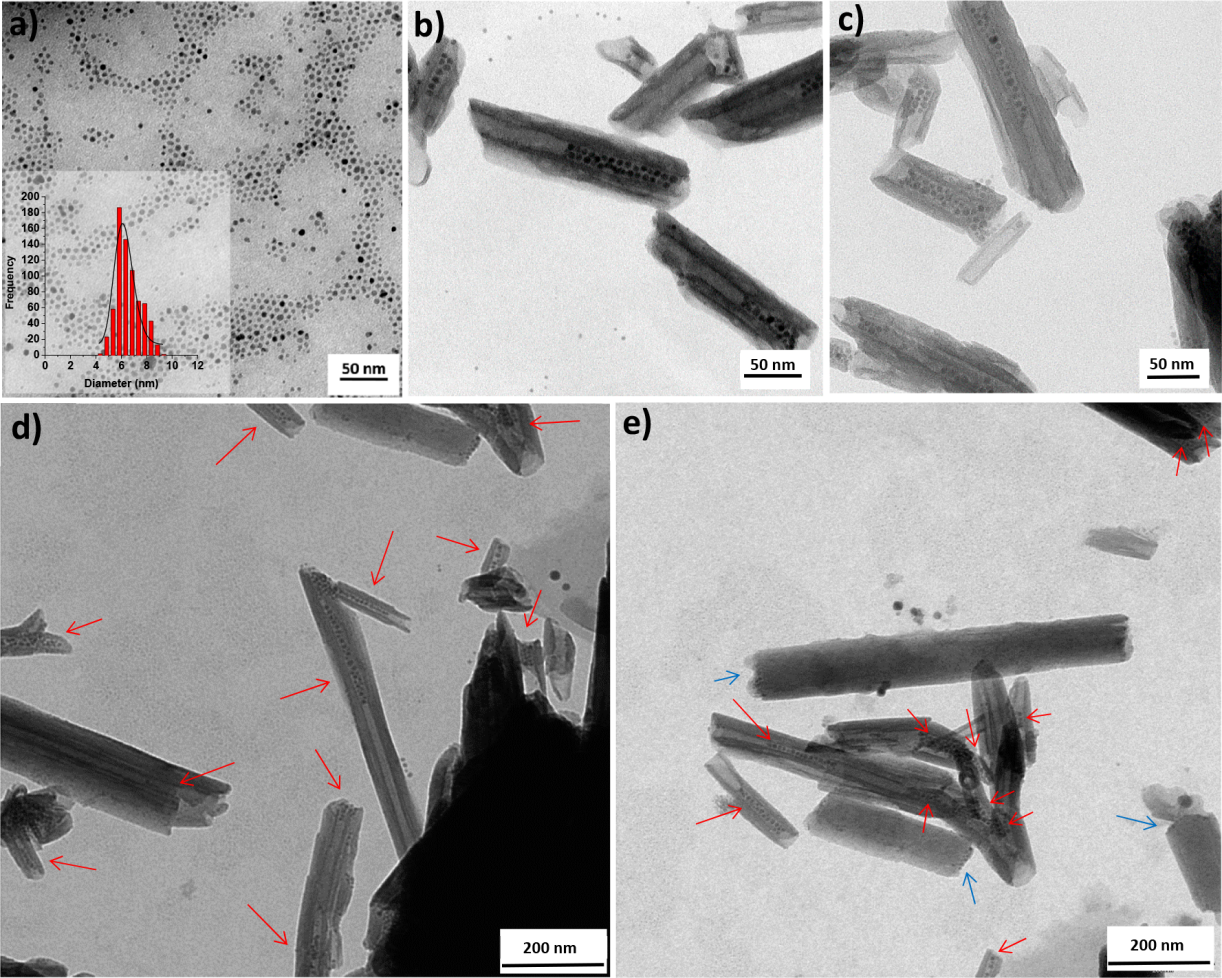
TEM micrographs of (a) as-synthesized SPION@OA (in the inset the SPION diameter distribution); (b–e) SPION-in-HNT prepared by prefunctionalization of HNT with TDP in the lumen. Panels (b) and (c) show images taken at higher magnifications compared to that used for (d) and (e). Red arrows mark the HNT containing SPION. Blue arrows indicate SPION at the edge of few HNT.

The preparation of SPION-in-HNT nanocomposite was achieved using SPION3@OA nanoparticles, obtained by the same thermal decomposition method^55^ used previously (see section 2.1), which this time afforded magnetic NPs with 6.1 ± 1.3 nm mean diameter as shown by TEM (Figure 3a) and a hydrodynamic diameter of 10.9. ± 3.0 nm (Figure S11).

The as-synthesized SPION@OA were effectively loaded into the HNT lumen by using repeated vacuum/N_2_ cycles under stirring while keeping the temperature at 0 °C with an ice bath to avoid the heavy evaporation of the volatile solvent. The procedure was repeated until the light brown color of the suspension (due to SPION@OA) turned transparent, and conversely, the deposited HNT turned from white to brown. The SPION loading was selectively directed into the inner part of HNT, without any relevant interaction with the HNT outer surface, as clearly shown by TEM analysis (Figure 3b–e). This result shows that the size and the nature of coating of SPION have a major role in successful loading into the inner lumen of HNT. Despite the fact that SPIONs used in the electrostatic approach (see above; section 2.1) were in the size range of 5.1–6.9 nm, which is less than the half of the inner lumen diameter, no loading was observed when they were coated with DMSA or the other polar molecules. On the contrary, SPION@OA were able to reach the inner lumen probably mostly due to the apolar weak interactions between the aliphatic chains on NPs (OA) and in the inner lumen of the nanotubes (TDP). TDP functionalization is also mandatory to observe the effective loading of SPION. Indeed, treating SPION3@OA with pristine HNT resulted in just sporadic loading into HNT, as clearly shown by TEM (Figure S12).

Unfortunately, the commercial pristine HNT used in this work were of low quality if compared with other HNT derived from other mines. In some images, we evidenced the presence of unrolled HNT or kaolin-like sheets (see Figure S13). In Figure 3e, there is a marked difference between the usual HNT, filled with SPION, that presents a lumen and walls clearly distinguishable (indicated with red arrows), and some peculiar HNT (highlighted with light blue arrows) that have a much larger diameter and do not clearly show the lumen, as if they are not empty, thus hampering the entrance of SPION. In this case, some SPION interacted preferentially with the HNT edges rather than with the lumen. Moreover, when we tried to completely fill all the HNT by doubling the SPION amount, we noticed by TEM analysis (Figure S14) that only a part concurred to increase the amount of SPION into HNT, the remaining been captured by the kaolin-like sheets.

### 2.4 Australian HNT with a Larger Lumen Filled with Apolar SPION@OA

**Figure 4:**
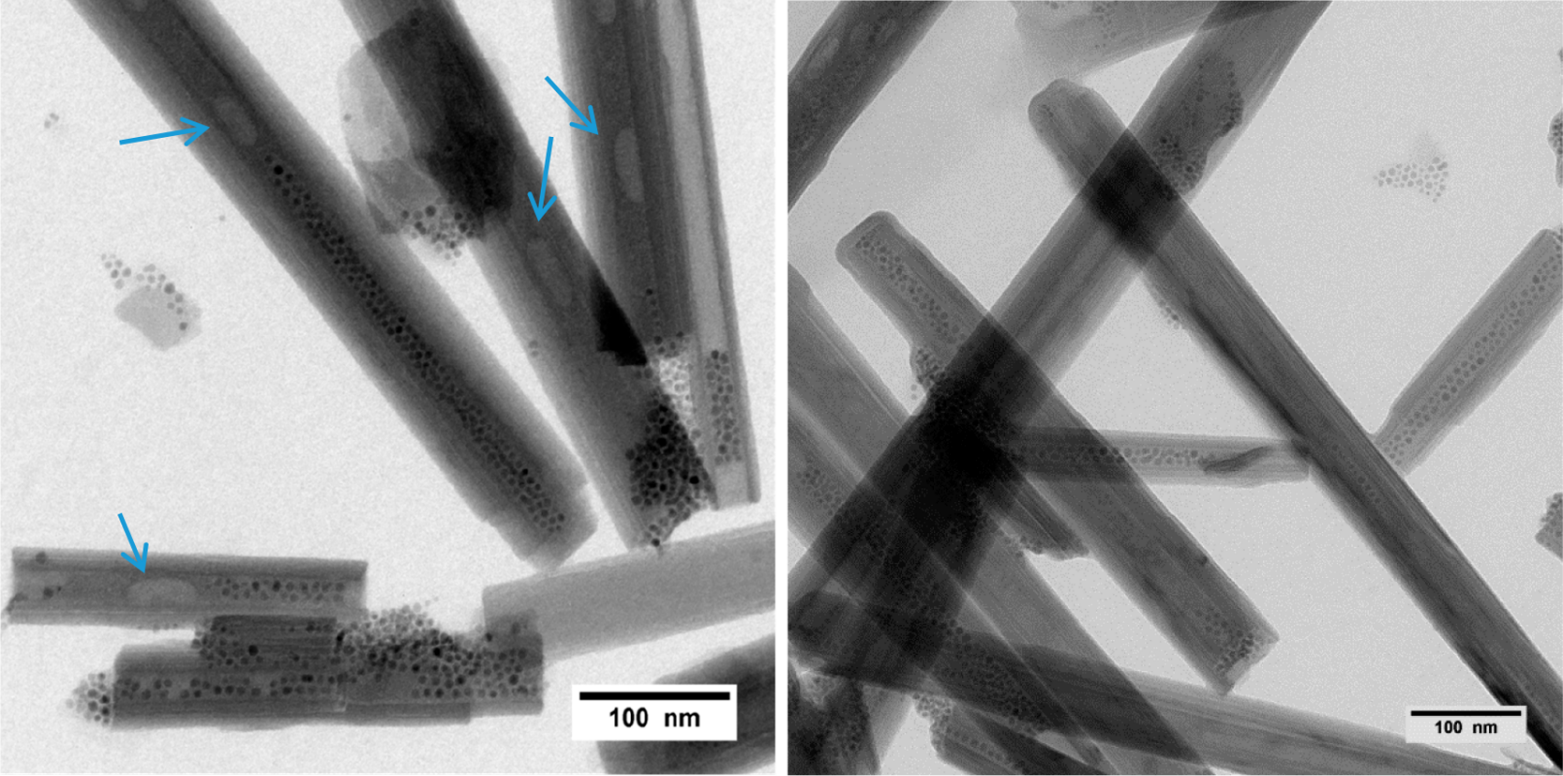
TEM micrographs of SPION-in-A-HNT (A-HNT = Australian Camel Lake halloysites). Blue arrows mark the HNT containing possible air bubbles.

Although very promising, the results obtained so far are not totally satisfying in terms of the amount of SPION embedded in the HNT. We ascribed this behavior to the poor quality of the commercial HNT batch, containing some large and apparently filled HNT. In order to verify this hypothesis, we considered a new sample of HNT derived from the Camel Lake mine (Australia). These HNT are characterized by a higher regularity than that of Aldrich HNT (Figure S15), with a larger lumen (23.8 ± 6.0 nm), even though they are on average longer (770 ± 300 nm). As in the case of the commercial HNT, we made the inner lumen of Australian HNT (A-HNT from now on) apolar by selective functionalization with TDP. Also in this case, the SPION@OA were loaded selectively within the A-HNT@TDP, but only when a maximum amount of 0.4 mg of SPION per 10 mg of HNT was used (Figure 4). As for the commercial HNT, the SPION did not completely fill the lumen. Nevertheless, when we tried to increase the SPION/HNT mass ratio, we found that SPION were massively localized also outside the lumen (Figure S16). At the same time, some semifull or apparently empty HNT were visible, although the lumen of the A-HNT was on average wider, such as not to prevent the entrance of NPs of 5–6 nm diameter. We think that the ability of air to escape from the HNT is essential to fully fill a nanotube. Indeed, in some TEM images air bubbles seem to be present within the inner lumen where SPION were not localized (see Figure 4, blue arrows). Moreover, to be more effective in the removal of air contained in the HNT lumen, we applied a prevacuum cycle to the HNT before adding the SPION. In that case, we did not find a significant number of particles in the lumen, but rather all were external to the HNT (Figure S17). This evidence made us conclude that the entry of SPION in the lumen takes place immediately after the break of the vacuum and the restoration of the ambient pressure in the reaction vessel.^48^ Obviously, the proper concentration of NPs must be present in order to make the loading as effective as possible.

### 2.5 Magnetic Properties Characterization of SPION-in-HNT Nanocomposite

**Figure 5:**
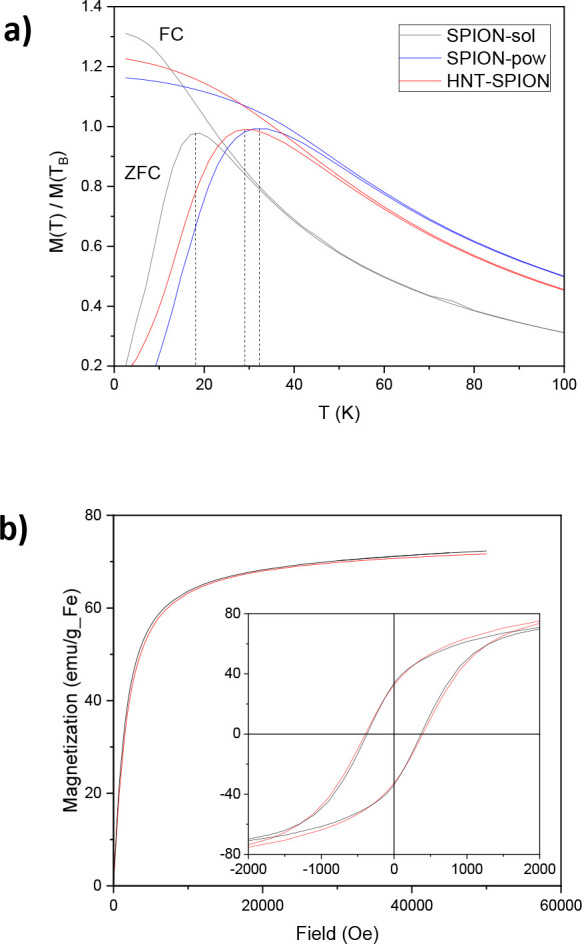
(a) ZFC (lower curve) and FC (upper curve) magnetizations acquired with a 50 Oe field. The vertical dashed lines remark the position of the ZFC maxima corresponding to the blocking temperatures (*T*_B_). For a better presentation, each curve was normalized to the magnetization value at the corresponding *T*_B_. (b) Magnetization curves at high (300 K, main panel) and low (2.5 K, inset) temperature for SPION (black line) and SPION-in-HNT (red line).

Magnetic measurements performed on SPION and SPION-in-HNT demonstrated that the nanoparticles display the typical superparamagnetic behavior expected for a set of nanoparticles of this size and that the main magnetic properties have been maintained after the SPION embedding in HNT. The pristine SPION were measured as a hexane solution (SPION-sol) and dried powder (SPION-pow). This last measurement was also used to estimate and remove the diamagnetic contribution of the solvent, thus obtaining reliable magnetization saturation (*M*_S_) value. The *M*_S_ of SPION was estimated as the magnetic moment recorded at the highest experimental field applied (50 kOe) divided by the iron content of SPION-sol evaluated by AAS analysis (2.2% w/w). As expected for systems of reduced size, the *M*_S_ value of the SPION (72 emu/g Fe at 300 K and 82 emu/g Fe at 2.5 K) is lower than the bulk value of magnetite, but it is quite high for 6 nm nanoparticles, where normally a considerable ratio of disordered spins at the surface to ordered spins in the core is observed.^64^ The ZFC/FC magnetization curves (Figure 5a) evidenced the superparamagnetic behavior of the samples, characterized by a maximum in the ZFC curve, the position of which determines as a rough approximation the blocking temperature (*T*_B_) of the system and by a thermal irreversibility at low temperature. It is interesting to note how the *T*_B_ of the pristine SPION increases from solution (18.8 K) to powder (32.3 K), while it reaches an intermediate value for SPION-in-HNT (29.3 K). The increase of *T*_B_ for a nanoparticle set measured in different conditions is generally ascribed to the enhanced interparticle interactions.^65^ In the cases of SPION-sol and SPION-pow, for instance, the dipolar interactions are stronger in the powder sample as the NPs are much closer each other with respect to the solution sample. Assuming that the embedding procedure of SPION-in-HNT induced only negligible modification in the magnetic anisotropy of the pristine SPION (see Figure 5a), the value of *T*_B_ observed for HNT–SPION suggests that the embedded nanoparticles are moderately interacting as between that of SPION-sol (low interactions) and SPION-pow (strong interactions), closer to the latter. This result is consistent with the expected confinement of the SPIONs in the HNT lumen that implies a reduction of the mean distance among the SPIONs with respect to a diluted solution.

As expected for a superparamagnetic system, the samples present magnetic hysteresis only for temperatures below *T*_B_, as shown in Figure 5b, where the magnetization curves, *M* versus *H*, are reported. At low temperature (2.5 K), both samples exhibit similar coercivity (*H*_C_ = 370 Oe for SPION and *H*_C_ = 395 Oe for SPION-in-HNT) and remanence (34 and 32 emu/g Fe, for SPION and SPION-in-HNT, respectively) that reduce to zero at high temperature (300 K). The similarity of the curve shape acquired before and after HNT embedding confirms that the loading procedure does not significantly alter the magnetic properties of the pristine SPION. The maintenance of the magnetic properties allowed us to estimate accurately the concentration of SPION in the SPION-in-HNT simply by the ratio of the saturation magnetization of the two samples. Actually, in our case, the comparison is very accurate, as the same ratio is found at all the magnetic fields because the whole normalized magnetization curves are perfectly superimposable.^66^ The iron concentration obtained by the superimposition of the curves at high temperature is 5.3% w/w, which is very close to the concentration that would be achieved if all the SPION in the preparation solution were loaded in the HNT lumen (5.5% w/w), corresponding to an encapsulation yield of 96% and a Fe_3_O_4_ content of 7.3% w/w. Furthermore, the total magnetization of the SPION-in-HNT is enough (3.82 emu/g) to guarantee a magnetic response to an appropriate magnetic gradient, allowing the use of the compound in several practical applications. This result underlines the efficacy of the proposed NP embedding procedure.

## 3 Conclusions

In summary, we have successfully prepared a novel magnetic halloysite nanocomposite with apolar SPION selectively loaded in the inner lumen of prefunctionalized HNT, exploiting vacuum-N_2_ cycles. The new SPION-in-HNT nanocomposite has been morphologically characterized by TEM as well as from the magnetic point of view. SPION@OA in the inner lumen of HNT did not change the magnetic properties, retaining the superparamagnetic character at 300 K with a moderate interparticle interaction once charged into the lumen of the nanotubes and a sufficient total magnetization such that the SPION-in-HNT obtained with this embedding procedure can respond to an external magnetic stimulus. The SPION-in-HNT nanocomposite was successfully obtained also using HNT of different source (A-HNT) endowed with a bigger lumen diameter.

Despite this more favorable characteristic for the NP loading, even in this case the NPs did not completely filled with all the HNT, suggesting that rather than the size of the nanoparticles it is the ability to release the initial air contained in the nanotubes to play a fundamental role in allowing the NP to enter.

Attempts to load preformed negatively charged SPION, exploiting the opposite charges of the HNT lumen, failed due to an early aggregation of the single NPs once subjected to an external force, which pushed them at the entrance of the lumen.

Finally, preliminary results on the inclusion of preformed Au nanoparticles of ≈5 nm coated with oleylamine (data not shown) suggest the procedure here proposed could be used as a general method for the loading of other kinds of inorganic nanoparticles stabilized with a hydrophobic layer. Depending on the nature of the loaded NPs the applications could span from catalysis to drug delivery or to wastewater treatment, just to mention a few.

## 4 Experimental Part

### 4.1 Materials and Instruments

Anhydrous FeCl_3_, FeCl_2_·4H_2_O (Merck Germany), ethanol 99.8% (Merck UK), oleic acid 90% (Alfa Aesar), *n*-hexane 96%, acetone (Scharlau), iron acetyl acetonate 97%, 1,2 hexadecandiol 90%, tetradecylphosphonic acid 97%, DMSA 98%, triethylamine 99%, 16-phosphonohexadecanoic acid 97%, protocatechuic acid 97%, gallic acid >97.5%, tetramethylammonium hydroxide pentahydrate 97%, sodium hydroxide pellets 98%, oleylamine 70%, diphenyl ether 99%, benzyl ether 98%, halloysite nanotubes HNT (Sigma-Aldrich), and Nile red >99% (Abcr) were all used as received without further purification. Toluene (99.5%, Sigma-Aldrich) was distilled from sodium under nitrogen prior to use. Ultrapure milli-Q water (Millipore, resistivity = 18 M Ω cm^–2^) was used for the preparation of the aqueous solutions.

ζ-potential measurements were carried out using a Malvern Zetasizer nano ZS instrument equipped with a 633 nm solid-state He–Ne laser at a scattering angle of 173°, typically dissolving samples at a concentration of 1 mg/mL or less at 25 °C. The measurements were averaged on at least three repeated runs.

Iron content of SPION was determined by different methods. AAS analysis was carried out on a PerkinElmer Pinaacle 900 instrument for SPION6@OA. For all the other preparations, a spectrophotometric method was employed on an Agilent model 8543 spectrophotometer at room temperature, using disposable cuvettes with 1.0 cm path length. For Fe determination by AAS, a few microliters of the particle suspension were digested with 1 mL of aqua regia/HCl overnight at room temperature in a 10 mL volumetric flask subsequently filled up with Milli-Q water. For spectrophotometric analyses, a few microliters of the particle suspensions were digested with 1 mL of aqua regia/HCl at room temperature in a 10 mL volumetric flask. Subsequently, they were filled up with (i) 0.1 mL of NH_2_OH solution (10% w/w), (ii) 6 mL of acetate buffer solution (pH 4.6, 0.15M), (iii) 0.2 mL of 1,10-phenantroline solution (0.6% w/w, H_2_O/MeOH 10:1), and (iv) Milli-Q water until a volume of 10 mL was reached. The absorbance of the band centered at 510 nm due to Fe(phen)_3_^2+^ complex was then measured, and the concentration of Fe was finally obtained, acquiring a calibration curve, which was obtained with standards prepared with the same procedure starting from a commercial AAS standard solution.

Thermogravimetric analysis (TGA) was carried out in air atmosphere and in the temperature range of 50–800 °C with a heating rate of 5 °C·min^–1^, using a Mettler-Toledo thermogravimetric balance (TGA/DSC 2 Star System) on ca. 10 mg of lyophilized samples.

ATR-FTIR spectra were acquired on a PerkinElmer Frontier instrument equipped with an ATR accessory with a diamond/ZnSe crystal. The IR spectra were registered between 4000 and 400 cm^–1^.

TEM micrographs were collected using a Zeiss Libra 200 FE instrument equipped with an in column prealigned omega filter that improves the contrast and a Schottky field-emission gun at 200 kV. Alternatively, TEM images were collected using an EFTEM LEO 912AB (Zeiss) at 100 kV. Samples were prepared by dropping a dilute solution of the samples onto 200 mesh carbon-coated copper grids, and after blotting the excess of water, the samples were let drying at least for 24 h in air. The nanoparticle size was measured by Pebbles and Pebbles-Juggler^68^ or by Image-J free software (Imaging Platform software, Olympus). For each sample, the NPs were measured at around 400.

Magnetic measurements were carried out by a SQUID magnetometer from Quantum Design Ltd. Powder samples (SPION-pow and SPION-in-HNT) were prepared by enclosing a small amount of powder in a Teflon tape; the solution sample (SPION-sol) was measured in a gel cap. The diamagnetic contribution of Teflon was found to be negligible, while that of the cap and solvent was evaluated as the magnetization component, linear with the magnetic field, needed to be added to the SPION-sol magnetization in order to obtain the same slope of the SPION-pow curve in the high-field region. The magnetization curves were obtained by acquiring the magnetic moment of the sample as a function of the applied magnetic field ranging from 0 to ±50 kOe, at low (2.5 K) and room temperature (300 K). The zero-field-cooled (ZFC) and field-cooled (FC) magnetizations were acquired as a function of the temperature applying a 50 Oe probe field after cooling the sample in the absence (ZFC) and presence (FC) of the probe field.

### 4.2 Synthesis of Magnetic Nanoparticles and Attempts for Their Loading in the HNT Lumen

SPION@OA and their derivatives after ligand exchange with DMSA, GA, PA, and TMAOH are described in the Supporting Information, together with the loading attempts of these negatively charged SPION-in-HNT and the one-pot procedure with thermal decomposition synthesis of SPION in the presence of HNT.

### 4.3 Modification of Halloysite Lumen with Tetradecylphosphonic Acid

Halloysites (250 mg) were added under stirring to a solution of TDP (0.278 g, 1 mmol) in 250 mL of 4:1 EtOH/H_2_O. The EtOH/H_2_O solution was adjusted to pH 4 with HCl 0.1 M. The halloysite suspension was transferred to a 500 mL flask, which was then evacuated using a vacuum-pump. The fizzing of the suspension indicated that air was removed from the halloysite lumen and replaced with TDP solution. The vacuum/nitrogen cycles were repeated 3 times in order to maximize TDP loading. After stirring for a week at room temperature, the modified halloysites were rinsed 5 times with EtOH/H_2_O, recovered by centrifugation, and finally dried at 100 °C overnight under vacuum. The TDP excess was recovered from the gathered supernatants and stored for the next functionalization process. After the functionalization process, the colloidal stability of HNT–TDP aqueous suspension was similar to that of untreated HNT at the same pH, indicating that the outermost surface of the clay nanotubes was not hydrophobized. Recovered HNT–TDP: ca. 250 mg.

### 4.4 Qualitative Assay for Lumen Hydrophobicity by Nile Red Dye

Nile Red (1 mg, 3.1 × 10^–3^ mmol) was dissolved in 10 mL of ethanol and left under stirring until the compound was completely dissolved. Then, HNT–TDP (20 mg) were added to the alcohol dye solution, and the flask was cooled at 0 °C before reducing the pressure by a mechanical pump and leaving the mixture for 2 h. The halloysites were recovered by centrifuging at 4226 rcf for 10 min. The blue-violet pellet was separated from the supernatant, washed with water, and recovered by centrifugation. After the HNT were dried, the recovered pellet appeared blue under visible light and were red-emissive under UV-lamp irradiation. The very same procedure was followed for the loading of Nile Red in pristine HNT. After washing the treated HNT with water, the color under visible-light irradiation was blue-violet, while under UV light irradiation, no light emission was detected.

### 4.5 Preparation of HNT–TDP with SPION Selectively Loaded in the HNT Lumen (SPION@OA-in-HNT–TDP)

In a Schlenk tube, SPION3@OA (3.75 mL, 2.2 mg/mL) were mixed with 150 mg of HNT–TDP in 150 mL of *n*-hexane. The suspension was shaken for 1 min by a vortex, then cooled at 0 °C, and while standing in the ice bath, it was placed under reduced pressure and magnetic stirring until the solvent was visibly reduced. Then, another 150 mL of *n*-hexane were added. The color of the supernatant turned clearer with respect to that in the beginning (from light brown to beige). The cycle of reduced pressure under stirring was repeated once more, until the supernatant appeared completely clear (about 3 h). The SPION@OA-in-HNT-TDP were recovered by centrifugation (3 min, 470 rcf). The supernatant was removed, and the precipitate was washed with 5 mL of *n*-hexane.

### 4.6 Pretreatment of the HNT Mineral to Obtain Purified HNT in Powder

A piece of mineral clay was cut into thin slices, keeping part of the clay as white as possible. Slices were dried at 50 °C for 1 h. Then, the slices were gently hand-ground in a mortar. To remove the soluble impurities, 2 g of powder was dispersed in 20 mL of distilled water, followed by sonication for 20 min, 20 min of stirring, then centrifugation at 117 rcf for 20 min. Then, the slurry was redispersed in 500 mL of distilled water after the pH was adjusted at ca. 7 to achieve a good suspension. The suspension was left to settle out the nondissolved impurities, and the supernatant containing HNT was transferred to another vial. The adjustment of pH to slightly acidic values (≈4–5) caused the flocculation of HNT. The supernatant was discarded, and the HNT slurry was dried at 60–80 °C and ground in a mortar to give a white powder ready for further uses.

## Supporting Information Available

Experimental procedures, DLS and ζ-potential measurements, TEM images for the various HNT samples obtained with the electrostatic procedure, TEM images for the HNT sample prepared by in situ procedure, digital picture of HNT samples treated with Nile Red, DLS measurements and TEM images of many assays carried out on HNT–TDP samples by using the premodification procedure (PDF)
