# Supplementary material for: An Approach for Magnetic Halloysite Nanocomposite with Selective Loading of Superparamagnetic Magnetite Nanoparticles in the Lumen
Source: Inorg Chem. 2020 Aug 12;59(17):12086–96. doi: 10.1021/acs.inorgchem.0c01039 (PMC8009513; doi:10.1021/acs.inorgchem.0c01039)
Supplement: Supplementary file 1 — ic0c01039_si_001.pdf [file ic0c01039_si_001.pdf]

An Approach for Magnetic Halloysite Nanocomposite with Selective Loading of Superparamagnetic Magnetite Nanoparticles in the Lumen

Hady Hamza,[a] Anna Maria Ferretti,[b] Claudia Innocenti,[c,d,e] Katarzyna Fidecka,[a] Emanuela Licandro,[a] Claudio Sangregorio,[c,d,e,*] Daniela Maggioni[a,d,*]

[a] *Dipartimento di Chimica, Università degli Studi di Milano, Via Golgi 19, 20133 Milano, Italy*

[b] *SCITEC-CNR, Sede Secondaria via G. Fantoli 16/15, 20138 Milano, Italy*

[c] *ICCOM-CNR, via Madonna del Piano 10, 50019 Sesto Fiorentino, Italy*

[d] *Consorzio INSTM, Via G. Giusti, 9, 50121, Firenze, Italy*

[e] *Dipartimento di Chimica, Università degli Studi di Firenze, via della Lastruccia 3, 50019 Sesto Fiorentino, Italy*

** Corresponding author: daniela.maggioni@unimi.it*

Syntheses of SPION@OA, ligand exchange processes, attempts for the loading of negative charged SPION in the lumen of HNT and one-pot loading attempt are described here following.

1 Synthesis of SPION1@OA by a coprecipitation method in a biphasic system with NaOH. The synthesis was carried out by following a slightly modified literature procedure.[1] Briefly, in a two-neck round-bottom flask, sodium oleate (4 mmol, 1.218 g), anhydrous $FeCl_3$ (1 mmol, 0.162 g), $FeCl_2 \cdot 4H_2O$ (0.5 mmol, 0.099 g) and NaOH (3mmol, 0.122 g) were dissolved in a mixture of deoxygenated water (by bubbling N_2 gas for 30 min, 3.0 mL), ethanol (3 mL) and toluene (5.25 mL) under nitrogen. The mixture was refluxed at 74 °C for 4 h. Then the final black mixture was left to return to room temperature by removing the heat source. The suspension was precipitated with ethanol and the sediment was isolated by magnetic decantation. The precipitate was dispersed in toluene and centrifuged for 10 min at 7142 rcf to remove all the undispersed residues. The black toluene dispersion was precipitated with ethanol again and the solvent was removed through magnetic separation. Finally, SPIONs were redisposed in 40 mL toluene to form stable colloids and stored under nitrogen at -23 °C for further uses. Fe content by UV-vis spectroscopy was 3.3 mg/mL.

2 Synthesis of SPION2@OA by a coprecipitation method in a biphasic system without NaOH. The synthesis was carried out by following the same procedure already reported previously, but in the absence of NaOH.[1] Briefly, in a two-neck round-bottom flask, sodium oleate (8 mmol, 2.44 g), anhydrous $FeCl_3$ (1 mmol, 0.162 g), and $FeCl_2 \cdot 4H_2O$ (0.5 mmol, 0.099 g) were dissolved in a mixture of deoxygenated water (by bubbling N_2 gas for 30 min, 2.25 mL), ethanol (3 mL) and toluene (5.25 mL) under nitrogen atmosphere. The mixture was refluxed at 74 °C for 4 h. Then, the work-up, the recovery and the final storage of the NPs was as for the NPs obtained for coprecipitation in a biphasic system in the presence of NaOH. Fe content by UV-vis spectroscopy was 4 mg/mL.

3 Synthesis of SPION3@OA by a thermal decomposition method. The synthesis was carried out by following a slightly modified literature procedure.[2] Birefly, in a two-neck round-bottom flask, $Fe(acac)_3$ (0.353 g, 1 mmol), 1,2-hexadecanediol (1.435 g, 5 mmol), oleic acid (0.952 mL, 3 mmol), and 0.987 mL of oleylamine (3 mmol) were dissolved in 10 mL phenyl ether by magnetic stirring for 10 min under a nitrogen atmosphere. After having removed the magnetic stirrer, the mixture was heated at 15 °C/s from room temperature to 200 °C and left at this final temperature for 35 min. Then, under a blanket of nitrogen, the mixture was further heated up to 265 °C at 15 °C/s and left at this temperature for 35 min. Finally, the black-brown mixture was naturally cooled by removing the heat source to room temperature. The so-obtained suspension was precipitated with ethanol (20 mL), then the black precipitate was separated via centrifugation (10 min at 4226 rcf), then it was dissolved in 20 mL n-hexane in the presence of oleic acid (0.05 mL) and oleylamine (0.05 mL). Centrifugation (10 min at

4226 rcf) was applied to remove any undispersed residue. The SPION@OA were then re-precipitated with 40 mL ethanol, centrifuged (10 min at 4226 rcf) to remove the solvent, then re-dispersed in 15 mL n-hexane and stored under nitrogen at -23 ^{O}C for further uses. Fe content by AAS was 2.2 mg/mL.

4 Synthesis of SPION4@OA by a thermal decomposition method. The synthesis was carried out by following a slightly modified literature procedure.[3] Briefly, in a two-neck round-bottom flask, iron-oleate (1.37 g, 1.53 mmol), 1,2-hexadecanediol (1.185 g, 4.596 mmol) and oleic acid (0.448 mL, 1.41 mmol) were dissolved in 7.14 mL diphenyl ether by magnetically stirring for 10 min under a nitrogen atmosphere. The mixture was degassed at 90 °C under vacuum and stirring for 2 h. The mixture was then heated at 10 °C/min from 90 to 260 °C, and left at this final temperature for 30 min under nitrogen. Finally, the black-brown mixture was rapidly cooled by removing the heat source, and water bath till reach to room temperature. The so-obtained suspension was precipitated with acetone (50 mL), and the black precipitate was separated via centrifugation (10 min at 4226 rcf). After that, it was dissolved in 10 mL n-hexane, re-precipitated with 50 mL ethanol, centrifuged (10 min at 4226 rcf), re-dispersed in 35 mL n-hexane, and finally stored under nitrogen at -23 ^{O}C for further uses. Fe content by UV-vis spectroscopy = 1.9 mg/mL.

5 Attempts to fill HNT with negatively charged SPION. We started preparing SPION1@OA[1] where OA stands for oleic acid, with a hydrodynamic diameter of 13.5 ± 4 nm (see DLS measurement reported in Figure S1), which was slightly smaller than the average inner HNT diameter.

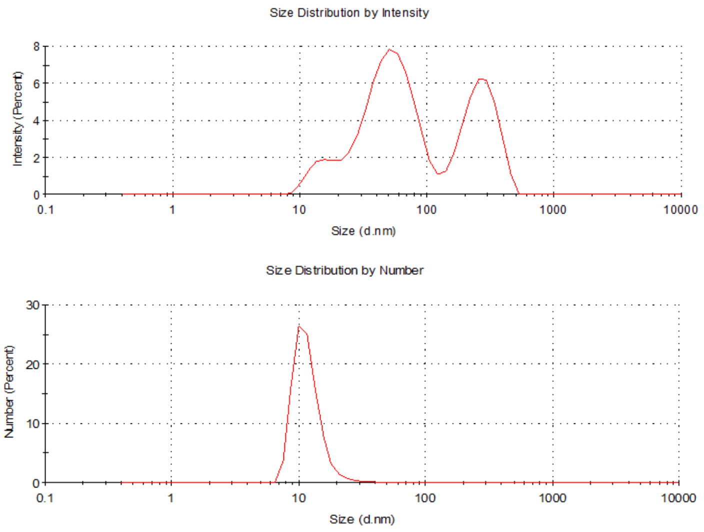

Figure S1. DLS of SPION1@OA suspended in n-hexane, synthesised by a coprecipitation method in a biphasic system and in the presence of NaOH. Top: Intensity-weighted diameter distribution, showing the presence of three distinct populations centred at 13.5 nm, 50 nm and 270 nm. Bottom: Number-weighted diameter distribution, showing that the large majority of the NPs belong to the smaller population, being the other two ascribable to sporadic aggregates.

In order to prepare negatively charged NPs, the oleate molecular layer was removed and substituted with a proper negatively charged stabilizing molecule. Hence, a first ligand exchange process was carried out with the bi-functional molecule dimercaptosuccinic acid (DMSA),[4] which is able on one side to bind to the surface and on the other side to self-deprotonate, giving rise to negatively charged as well as water-compatible SPION. The ligand exchange possibly needs to occur without NP aggregation, hence maintaining the hydrodynamic size observed on the starting SPION stabilized by OA. After the ligand exchange step, the SPION1@DMSA showed a hydrodynamic diameter suitable for the HNT loading (see Figure S2a).

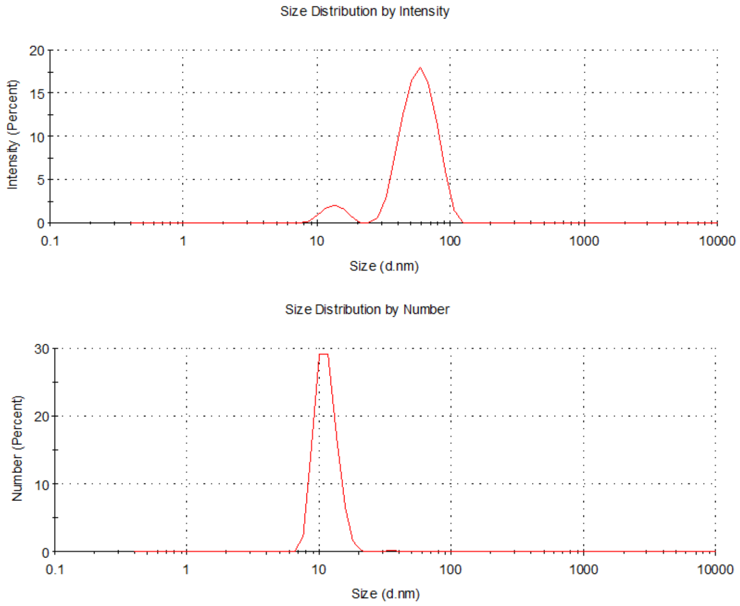

Figure S2a. DLS of SPION1@DMSA suspended in milliQ water after ligand exchange from SPION1@OA. **Top**: Intensity-weighted diameter distribution, showing the presence of two distinct populations centred at 13.5 nm and 60 nm. **Bottom**: Number-weighted diameter distribution, showing that the large majority of the NPs belong to the smaller population, being the other ascribable to sporadic aggregates of the main single NPs.

The loading tests were then carried out bringing the starting pristine-halloysite suspension to acidic pH (~3-4) in order to maximize the charge difference between the inner part of HNT (still enough positive at this pH) and the magnetic nanoparticle (still largely negative). However, both sonication and/or applied vacuum/N_2 cycles - to induce the air bubbles naturally present in the halloysites to come out, as well as the NPs of opposite charge to enter in the lumen - were not effective, and SPION were not found in the HNT inner part by TEM investigation. On the contrary, they always formed aggregates accumulating outside the nanoclay, and in some cases interacting with the HNT edges only (Figure S2b).

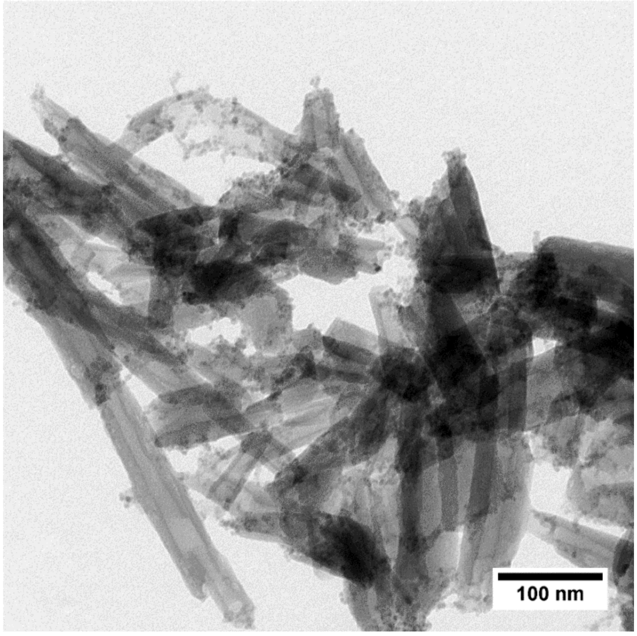

Figure S2b. TEM micrograph of pristine HNT treated with SPION1@DMSA in water suspension by vacuum/nitrogen cycles and sonication.

We then considered decreasing the size of the SPION by adopting different synthetic approaches for SPION2@OA (with a hydrodynamic diameter of 10.6 ± 4 nm,[1] see DLS size distribution in Figure S3) and SPION3@OA (with a hydrodynamic diameter of 8.0 ± 1.9 nm, see DLS size distribution in Figure S4a, and a mean diameter by TEM of 5.1 ± 1.6 nm,[2] Figure S4b).

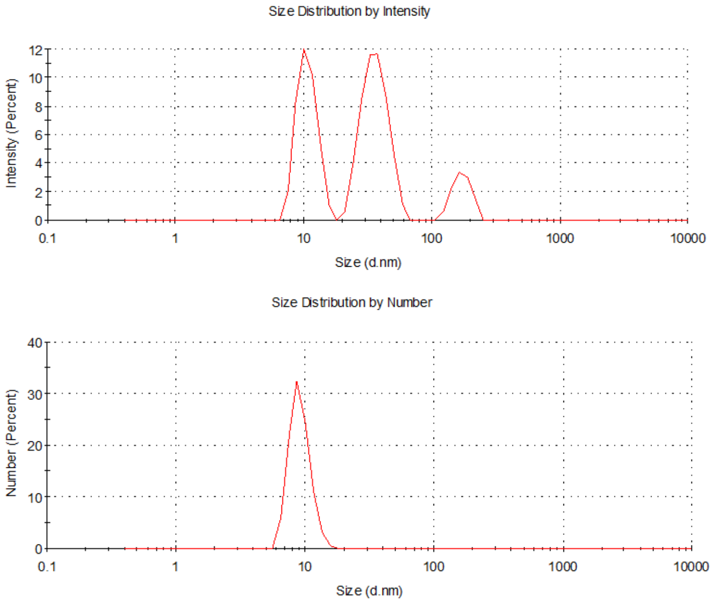

Figure S3. DLS of SPION2@OA suspended in n-hexane, synthesised by a coprecipitation method in a biphasic system without NaOH. **Top**: Intensity-weighted diameter distribution, showing the presence of three distinct populations centred at 10.6 nm, 35 nm and 150 nm. **Bottom**: Number-weighted diameter distribution, showing that the large majority of the NPs belong to the smaller population, being the other two due to sporadic aggregates.

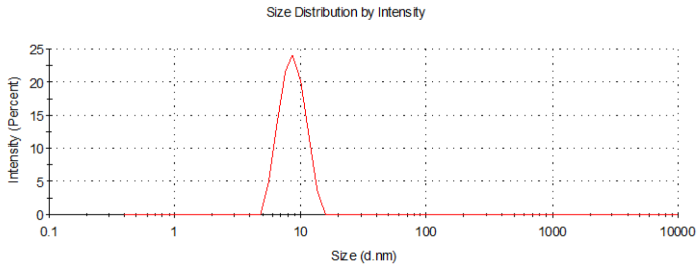

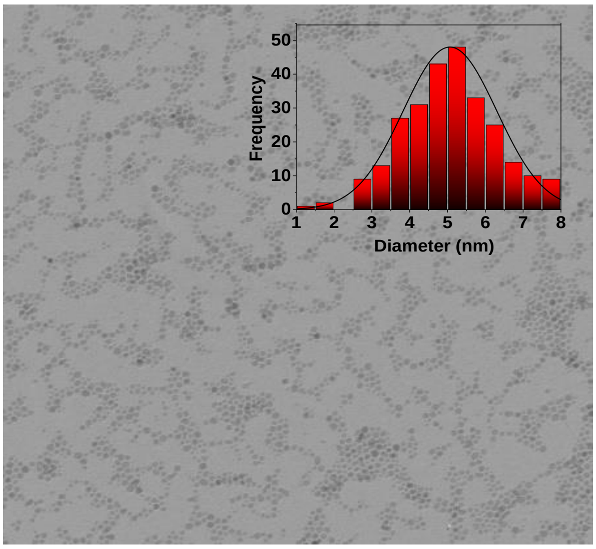

Figure S4. Top: Intensity-weighted diameter distribution by DLS measurements on SPION3@OA suspended in n-hexane, synthesised by a thermal decomposition method. **Bottom:** TEM micrograph of the same sample. In the inset it is reported the size distribution derived by Image-J software.

In both cases, as already previously done on bigger SPION, the oleate layer onto NPs was exchanged with DMSA. It is noteworthy that many times the ligand exchange OA/DMSA leads to the formation of aggregates, and the pH of the exchange process played a fundamental role. Indeed, if the ligand exchange is carried out in basic conditions (pH 9-10), the final colloidal water dispersions are much more stable, indicating that a more effective exchange is occurring. In any case, even when the hydrodynamic diameter was small enough, the negatively charged NPs were not able to enter into the HNT lumen (Figure S5).

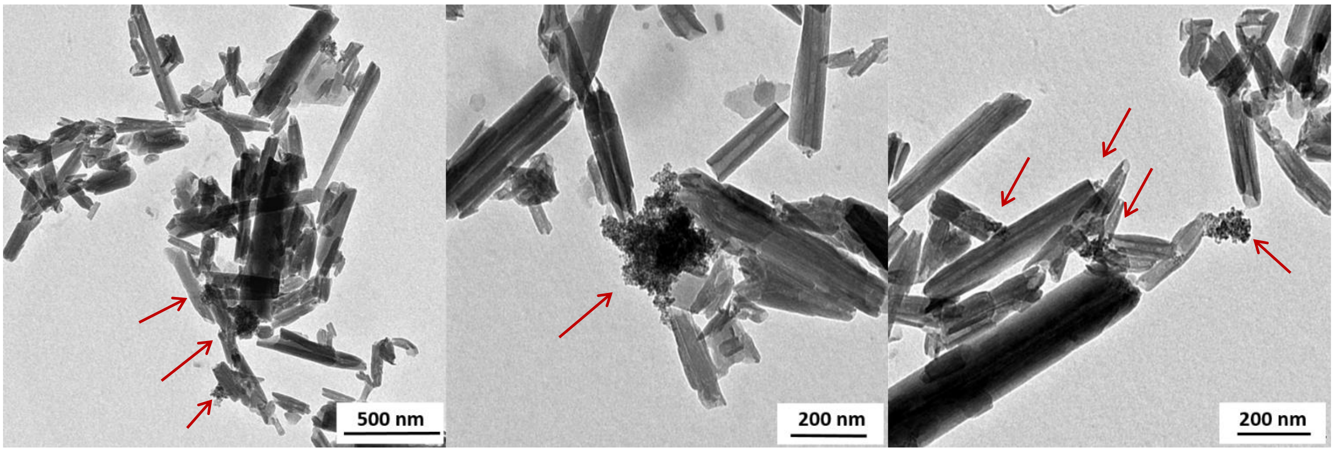

Figure S5. TEM images of pristine HNT treated with SPION3@DMSA in water suspension by vacuum/nitrogen cycles. Red arrows indicate the sites of SPIONs accumulation.

To explain the unsuccessful loading process, the hypothesis is that the DMSA could induce early aggregation of SPION during the reduced pressure/N_2 process, especially at acidic pH values. DMSA could form S-S bridges or hydrogen bonds between DMSA molecules lying onto different SPION, especially when they are pushed to stay in a small volume. Thus, we changed the type of hydrophilic coating, employing other bi-functional molecules, i.e. gallic acid (GA),[5] 16-phosphonohexadecanoic acid (PDA) and protocatechuic acid (PA) or the peptizing agent tetramethylammonium hydroxide (TMAOH)[6] (see Scheme S1). Except of PDA that did not give good suspensions after the ligand exchange, all the other molecules tested (see Scheme 2) effectively displaced the oleate molecules from the NP surface, giving rise to stable water colloids (see Figures S6-S8 for DLS measurements and Figures S5 and S7 for TEM images of SPION4@GA and SPION4@PA, respectively). Nevertheless, in no case we did get the loading in the HNT inner.

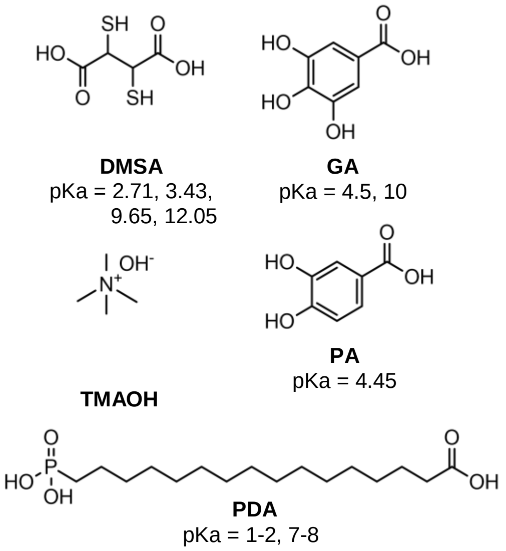

Scheme S1. Scheme of bifunctional ligands used for the OA exchange on SPION with their pK_a.

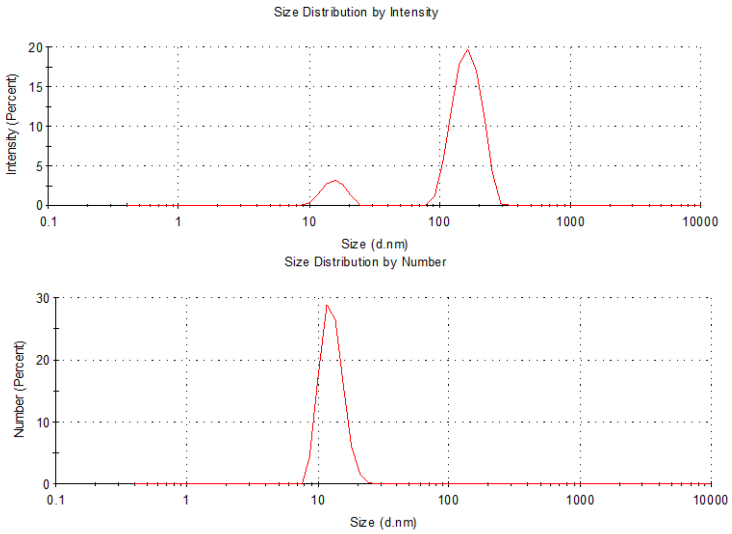

Figure S6a. DLS of SPION4@GA SPIONs suspended in MilliQ water after ligand exchange. **Top**: Intensity-weighted diameter distribution, showing the presence of two distinct populations centred at 13.0 nm and 160 nm. Bottom: Number-weighted diameter distribution, showing that the large majority of the NPs belong to the smaller population, being the other ascribable to sporadic aggregates of the main single NPs.

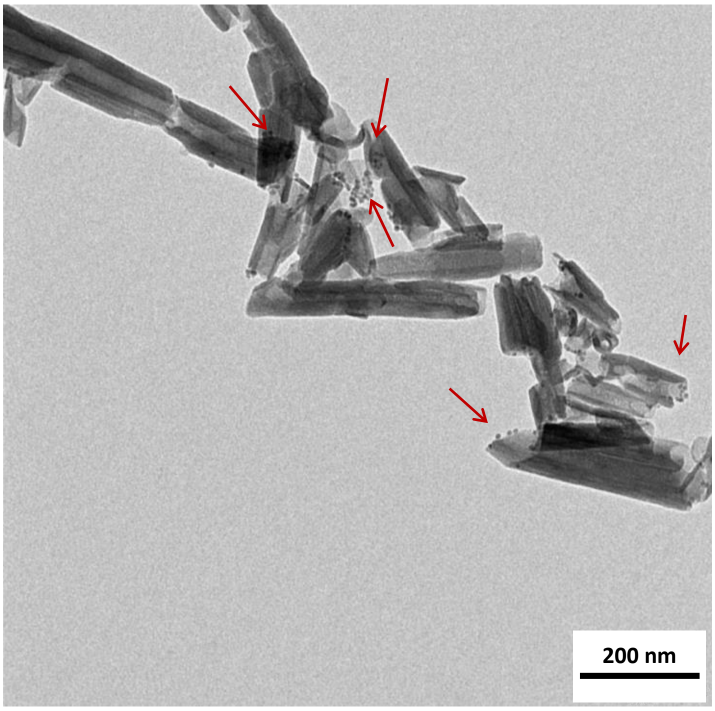

Figure S6b. TEM micrograph of pristine HNT treated with SPION4@GA in water suspension by vacuum/nitrogen cycles. Red arrows indicate the sites of SPIONs accumulation.

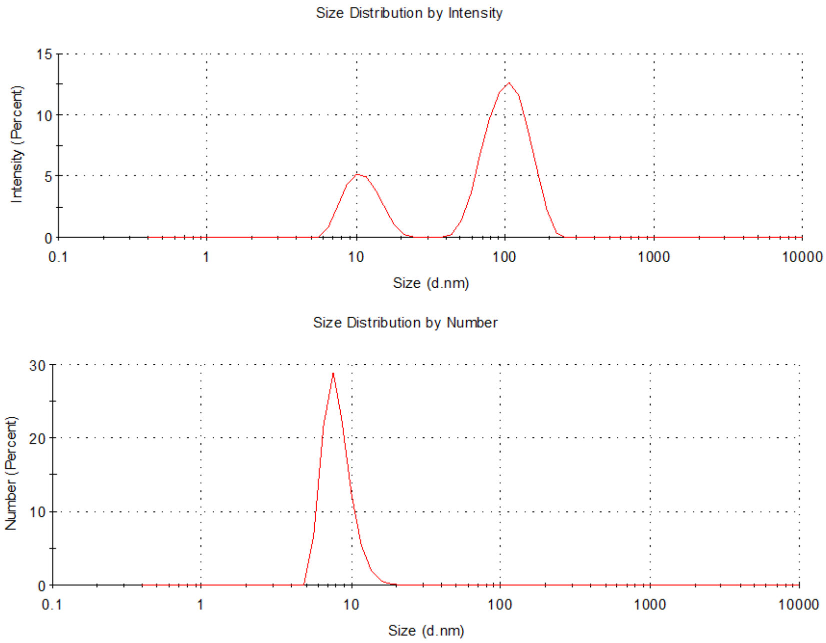

Figure S7. DLS of SPION4@TMAOH suspended in MilliQ water after ligand exchange. **Top**: Intensity-weighted diameter distribution, showing the presence of two distinct populations centred at 10.0 nm and 100 nm. Bottom: Number-weighted diameter distribution, showing that the large majority of the NPs belong to the smaller population, being the other ascribable to sporadic aggregates of the main single NPs.

Contrary to what observed for the other samples of SPION covered with DM SA, GA and PA, in the case of SPION4@TMAOH loading test, we did not observe any change in color of the recovered powders as well as any magnetic attraction when an external magnet was applied, and no TEM image was acquired.

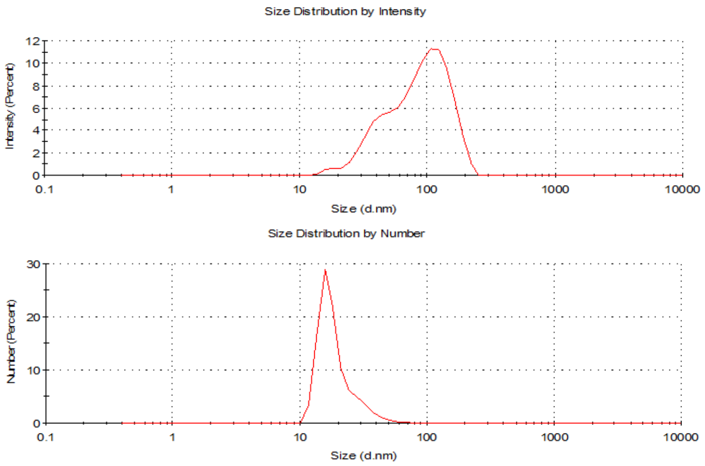

Figure S8a. DLS of SPION4@PA suspended in milliQ water. **Top:** Intensity-weighted diameter distribution, showing the presence of three distinct populations centred at 13.0 nm, 40 nm and 102 nm. Bottom: Number-weighted diameter distribution, showing that the large majority of the NPs belong to the smaller population, being the other two ascribable to sporadic aggregates.

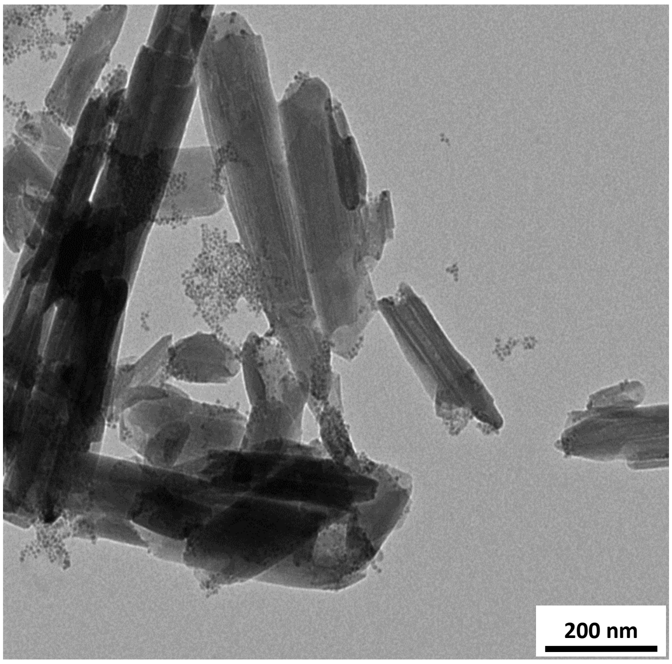

Figure S8b. TEM micrograph of pristine HNT treated with SPION@PA in water suspension by vacuum/nitrogen cycles.

5.1 Ligand exchange OA/DMSA (SPION1@DMSA, SPION2@DMSA, and SPION3@DMSA). The ligand exchange procedure was carried out following a procedure developed by some of us.[4] A sample of SPION@OA suspended in n-hexane containing a total amount of 5 mg Fe (1.5 mL SPION1, 1.25 mL SPION2, and 2.3 mL SPION3) was treated with 4 mL of acetone and centrifuged (4 min 7197 rcf) to remove the excess of oleic acid. The nanoparticles were re-suspended in 9 mL n-hexane and transferred in a Schlenk tube under nitrogen atmosphere. To this suspension a solution of DMSA dissolved in acetone (50 mg in 9 mL) was added, followed by 15 µL of triethylamine (TEA). The suspension in the Schlenk tube was then moved to an ultrasonic bath and the reaction mixture was kept at 54 °C for 40 min, maintaining the suspension under nitrogen atmosphere for the whole sonication time. The SPION were then collected with a magnet, re-suspended in 20 mL of acetone and centrifuged for 10 min at a rate of 7197 rcf. The last wash was carried out by re-suspending the pellet in 20 mL of milliQ water and centrifuged again (30 min, 7197 rcf). The washed SPION were finally re-suspended in 10 mL of milliQ water and stored under nitrogen atmosphere at 4 °C or at room temperature for further uses.

5.2 Ligand exchange OA/GA (SPION4@GA).[5] A sample of SPION@OA suspended in n-hexane (2.6 mL, 5 mg/mL Fe) was re-dispersed in 10 mL toluene. Gallic acid (GA) (43.2 mg, 0.254 mmol) was dissolved in a mixture of 110 µL pyridine and 322 µL toluene under 5 min sonication. Then, GA solution was added dropwise into SPION suspension, and the SPIONs precipitated immediately. The SPION@GA were then recovered by a permanent magnet and the supernatant was removed. The magnetic pellet was then re-suspended in 10 mL of 1% sodium carbonate solution, then SPION@GA were washed by adding an excess of ethanol (20 mL) and collecting the precipitating SPIONs by centrifugation (7197 rcf, 2 min). The pellet was then re-suspended in 3 mL water, precipitated again by adding ethanol and collected by centrifugation (7197 rcf, 2 min). Then, the resulting SPION@GA pellet was easily re-dispersed in 10 mL water, and stored at 4 °C for further uses. The same procedure has been used for the exchange OA/PA.

5.3 Ligand exchange OA/TMAOH (SPION4@TMAOH).[6] A sample of SPION@OA was suspended in n-hexane (2.6 mL, 5 mg/mL Fe) and dried under N_2 stream in a glass vial. The solid residue was treated with an aqueous solution TMAOH·5H_2O (1.25 mL 1.1 M) and ultra-sonicated at 54 °C for 40 min for re-disperse the SPION@TMAOH. After sonication, the suspension was diluted with 10 mL MilliQ water and further sonicated affording a clear brown suspension (final concentration of Fe_3O_4@TMAOH 0.5 mg /mL).

5.4 Attempts to load SPION@DMSA into HNT lumen. Water suspended SPION@DMSA (0.5 mg/mL) at pH 3-4 was mixed with 1 mL of HNT water suspension (10 mg) at the same acid pH value. The mixture was sonicated for 15 min, shaken on a vortex for 10 min, and then the suspension subjected

to reduced pressure (30 mmHg) for 30 min at room temperature. The sonication vacuum process was repeated for 5 times, giving rise to a brown HNT adduct affected by an external magnet, which was recovered by centrifugation (3 min, 470 rcf). The same procedure was used for the attempts of loading of the other SPION (SPION@GA, SPION@PA, SPION@TMAOH).

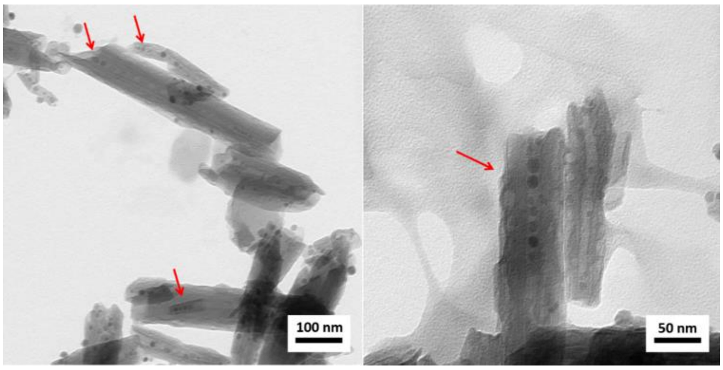

Figure S9. TEM images of HNT-SPION adduct obtained by thermal decomposition of iron precursor in the presence of HNT at high temperature and preceded by a vacuum/nitrogen cycle. Red arrows mark the SPION grown in the inner lumen of HNT.

6 One-pot procedure with thermal decomposition synthesis of SPION in the presence of HNT. The synthesis was carried out by following a slightly modified literature procedure, [2] and in the presence of HNT. Birefly, in a two-neck round-bottom flask, under nitrogen atmosphere, Fe(acac)$_3$ (0.353 g, 1 mmol) and HNT (0.706 g) were added to benzyl ether (10 mL) such that the mass ratio Fe: HNT was 1:2. The mixture was magnetically stirred for 10 min, then sonicated for 15 min and subjected to a reduced pressure (~ 1-2 mmHg) under stirring for 30 min. The sonication-vacuum-N_2 cycle was repeated for 2 times more. Finally, 1.435 g of 1,2-hexadecanediol (5 mmol) together with 0.952 mL of oleic acid (3 mmol) and 0.987 mL of oleylamine (3 mmol) were added and mixed under inert atmosphere. The magnetic stirrer was removed and the mixture heated from room temperature to 200 °C at a heating rate of 15 °C/s. Then, the mixture was left at 200 °C for 2 h, and then further heated up to 300 °C at the same rate as before and left at this final temperature for 1 h. At the end of the heating period the black-dark brown mixture was left to cooling down naturally to room temperature by removing the heat source. The so-obtained suspension was precipitated with ethanol (20 mL), then the black precipitate was recovered by centrifugation (10 min at 4226 rcf). The precipitate was re-suspended in 20 mL n-hexane adding also 50 µL oleic acid and 50 µL oleylamine. Centrifugation (10 min at 4226 rcf) was applied to remove possible free SPION or SPION externally interacting with the HNT. The SPION-in-HNT product was treated with ethanol, and centrifuged again (4226 rcf, 10 min) to remove all the high-boiling solvent or the surfactant excess. Then the solid was re-dispersed in 35 mL n-hexane and left under nitrogen atmosphere. After 2 days of decantation, the black supernatant

containing free SPION@OA was carefully removed. The solid residue was dried under vacuum and grinded, thus obtaining a grey and magnetic precipitate.

7 Attempt to Load SPION into unmodified pristine HNT lumen (SPION@OA-in-HNT). In a Schlenk tube a sample of SPION3@OA (0.5 mL, 2.2 mg/mL) were mixed with 20 mg HNT in 20 mL n-hexane. The suspension was shaken for 1 min by a vortex, then it was cooled at 0 °C, and while standing in the ice bath, it was placed under reduced pressure and magnetic stirring until the solvent was visibly reduced. Then, another 20 mL n-hexane was added. After 1 h of the cycle reducing pressure, the brown color of the supernatant due to the magnetic NPs remained unchanged with respect to the starting color. The HNT/SPION were recovered by centrifugation (3 min, 470 rcf), the supernatant removed and the precipitate washed with 5 mL n-hexane.

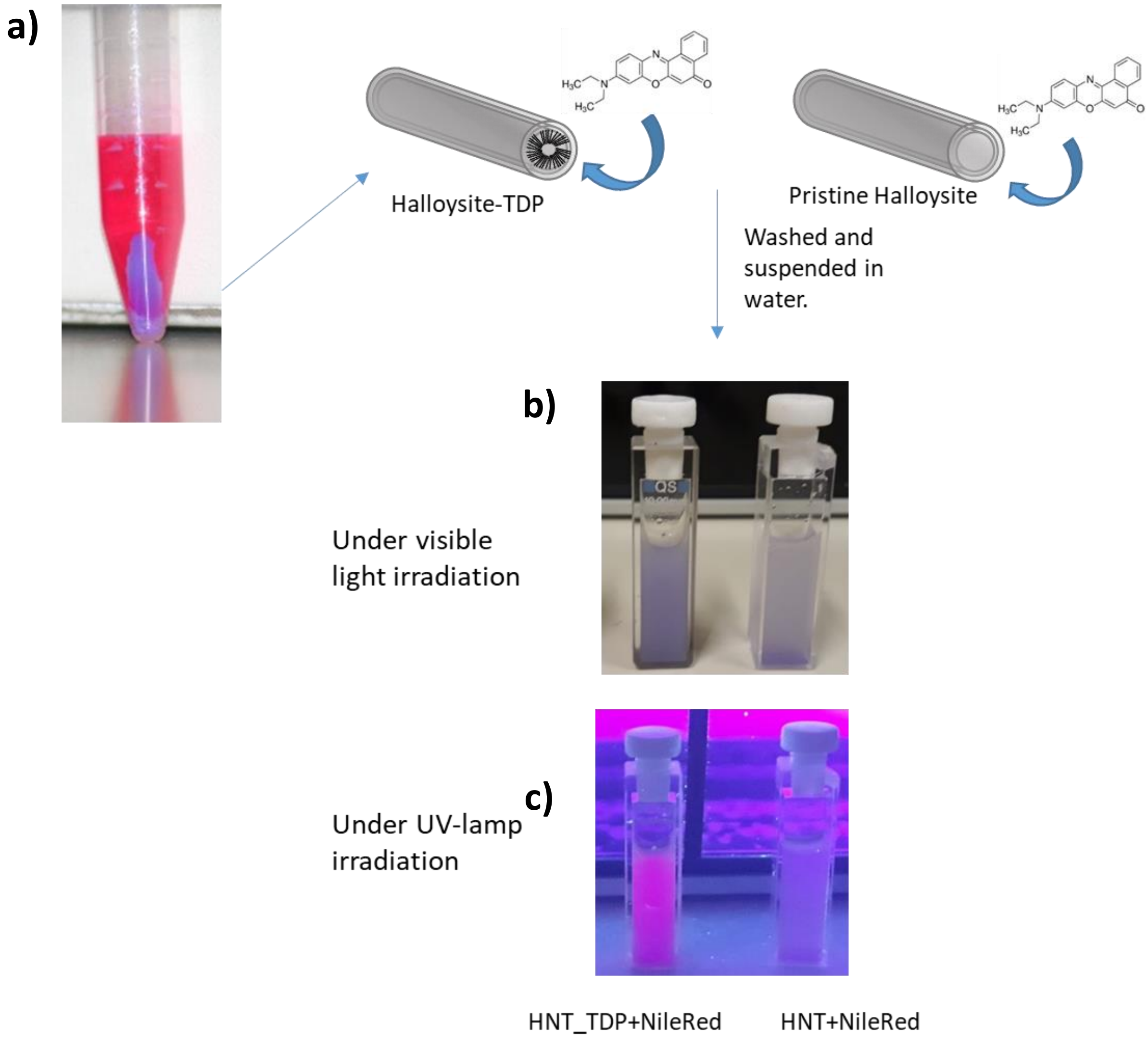

Figure S10. Digital pictures showing (a) the HNT interacted with an ethanol solution of Nile Red dye; (b) HNT-TDP (left) and pristine HNT (right) treated with Nile Red and irradiated by visible light; (c) the same samples of panel (b) irradiated by UV-light.

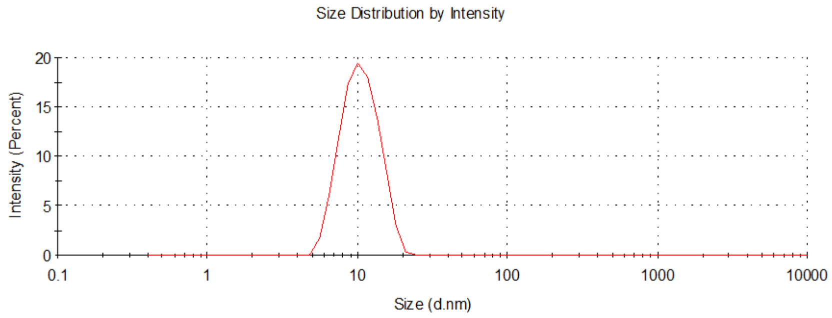

Figure S11. DLS of SPION3@OA suspended in n-hexane, synthesised by thermal decomposition and then used for the loading in the HNT-TDP.

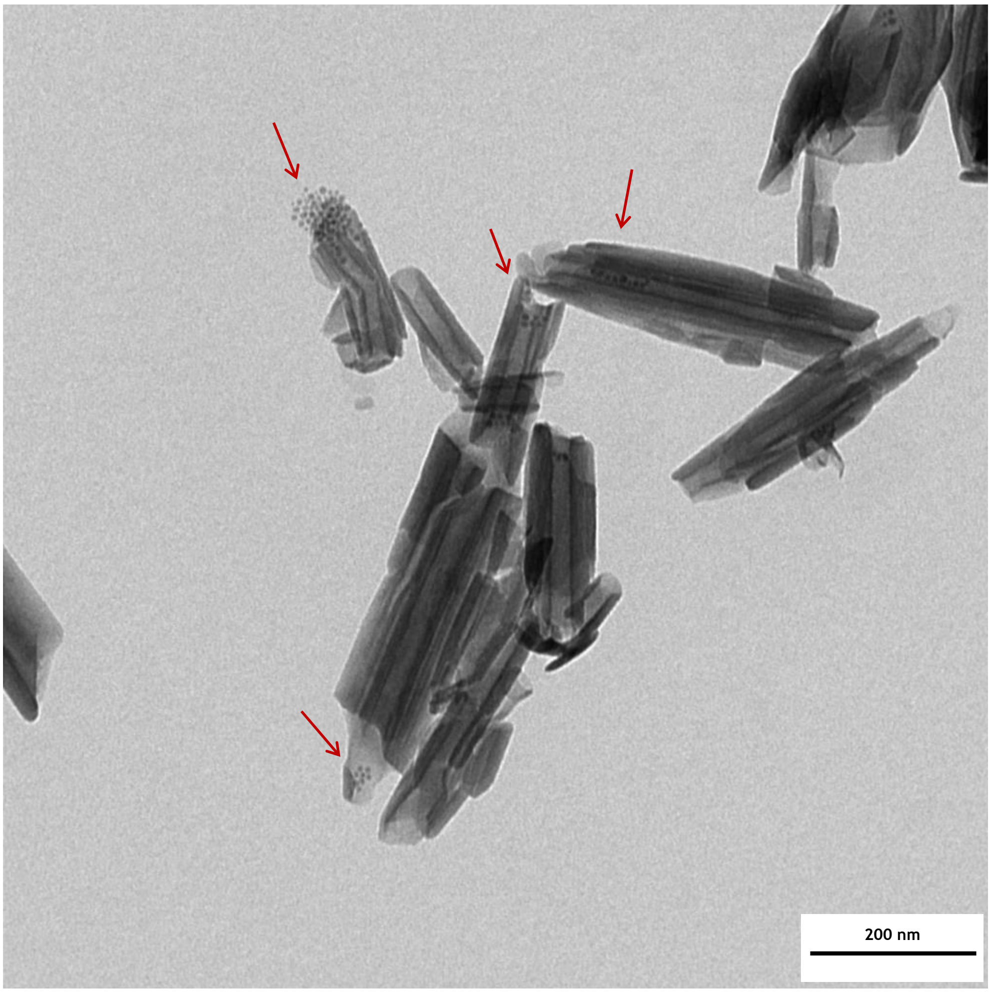

Figure S12. TEM micrograph of pristine HNT treated with SPION3@OA in n-hexane and with several repeated vacuum cycles. Red arrows indicate the sites of SPIONs accumulation.

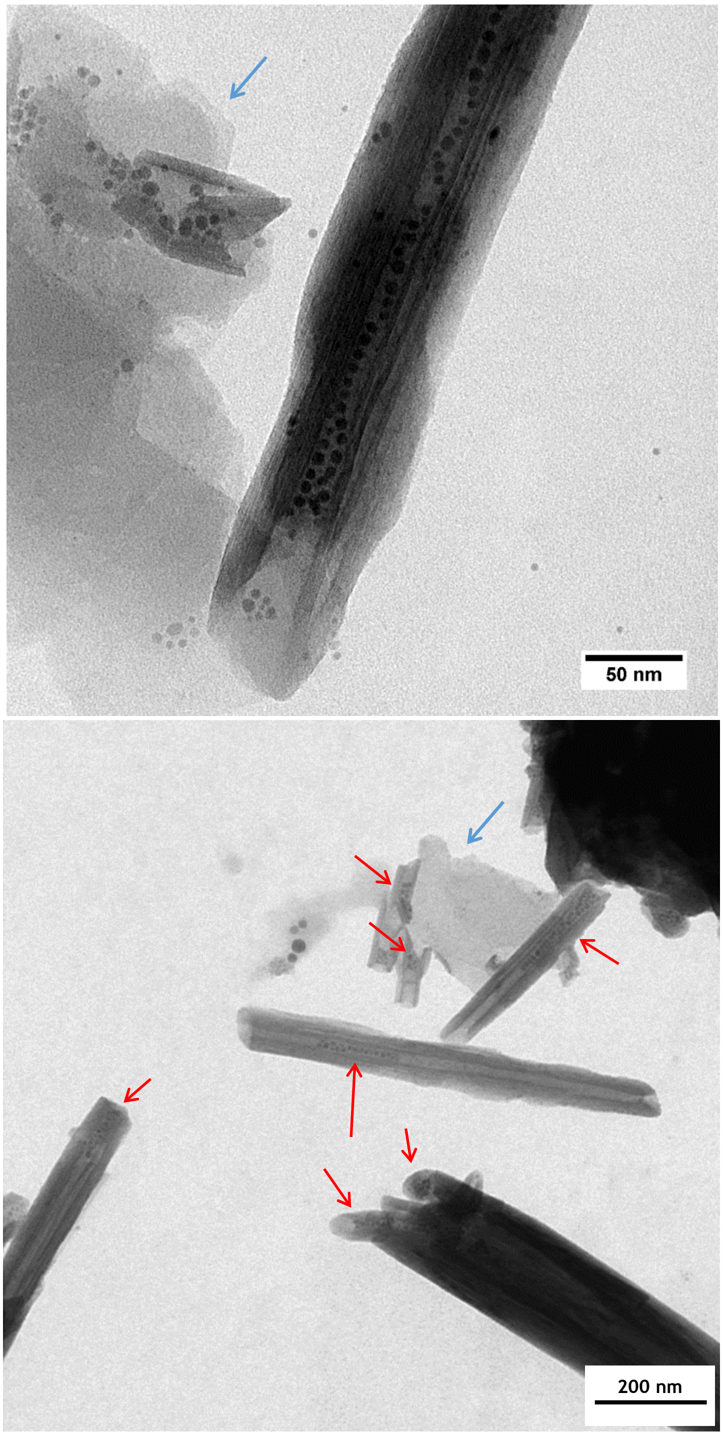

Figure S13. TEM micrographs of HNT-TDP treated with SPION3@OA in n-hexane and with several repeated vacuum cycles. Red arrows indicate the sites of SPIONs accumulation, while the light blue arrows indicate kaolin-like sheets.

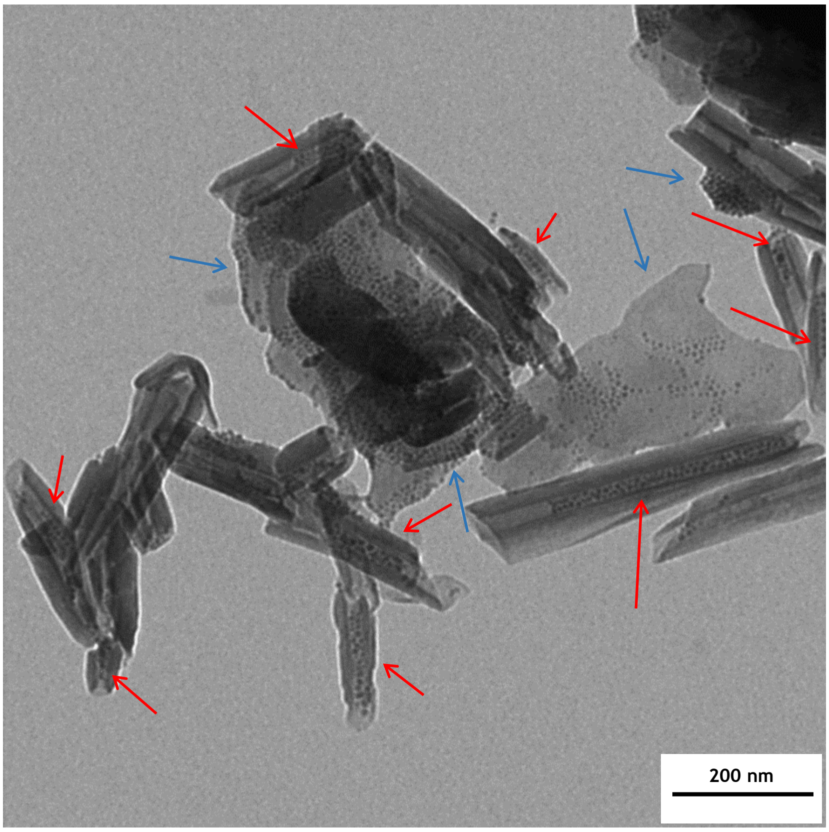

Figure S14. TEM micrographs of HNT-TDP treated with a double amount of SPION3@OA in n-hexane and with several repeated vacuum cycles. Red arrows indicate the sites of SPIONs accumulation, while the light blue arrows indicate kaolin-like sheets.

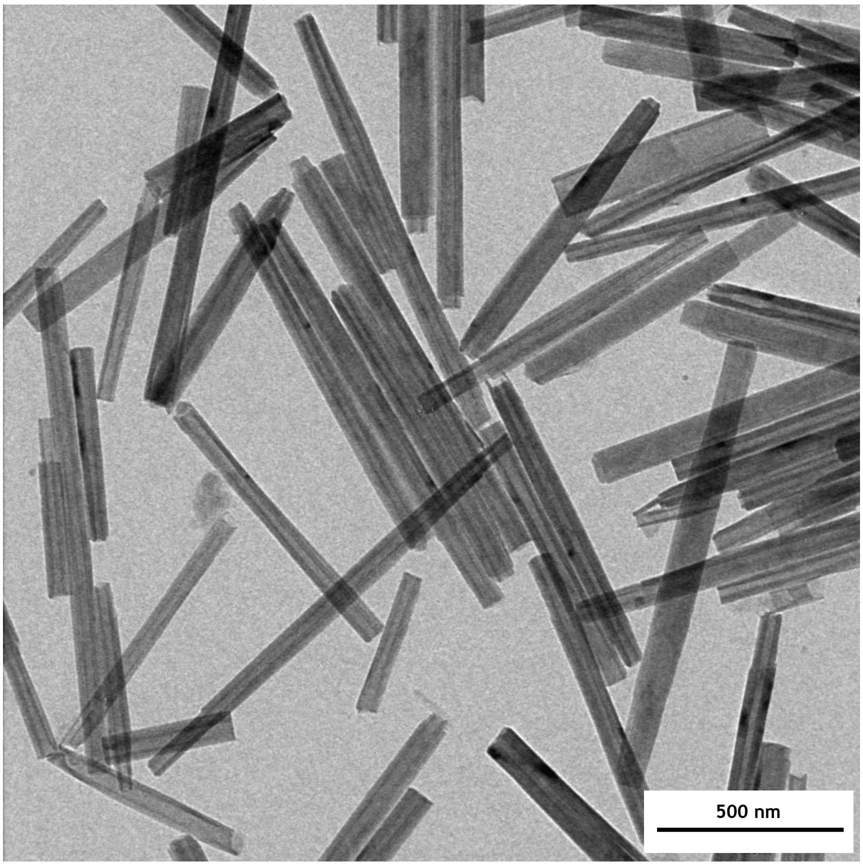

Figure S15. TEM micrographs of A-HNT (Australian HNT).

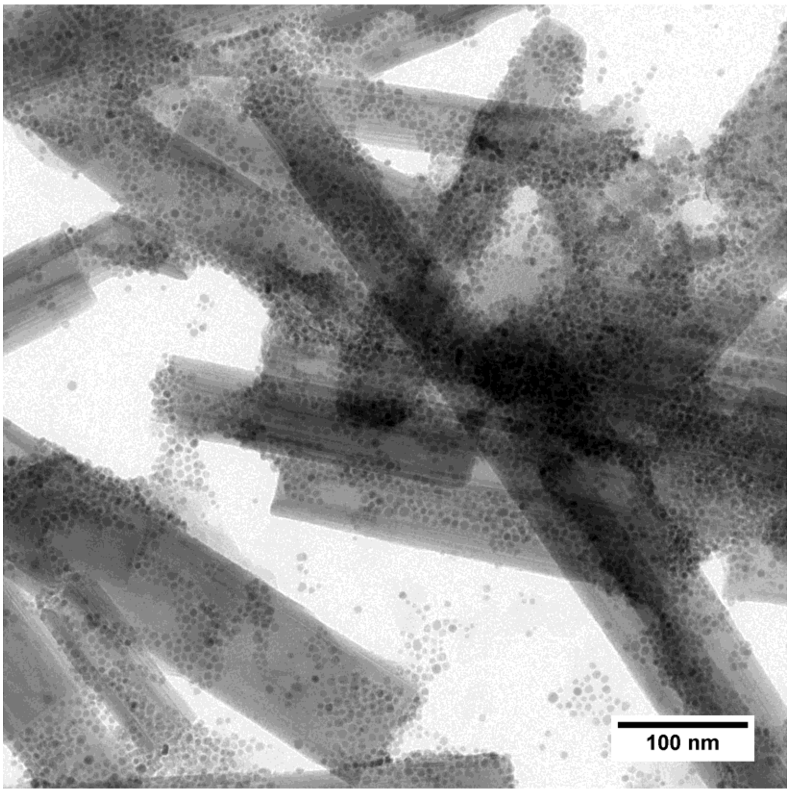

Figure S16. TEM micrographs of A-HNT-TDP treated with a double amount of SPION3@OA in n-hexane and with several repeated vacuum cycles.

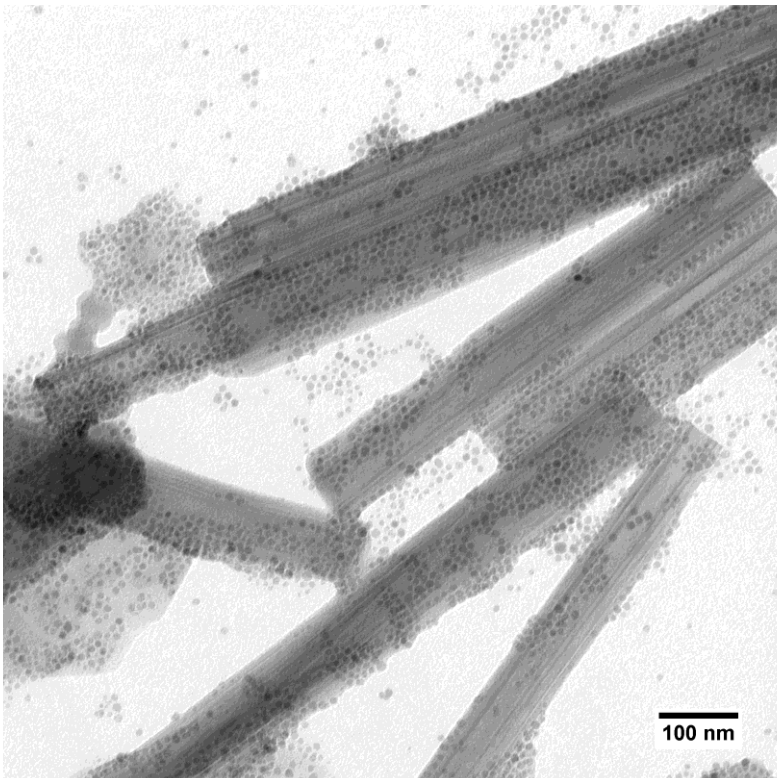

Figure S17. TEM micrographs of A-HNT-TDP subjected to a pre-vacuum-nitrogen cycle and then treated with SPION3@OA in n-hexane and with several repeated vacuum cycles.

References

(1) Jiang, W.; Wu, Y.; He, B.; Zeng, X.; Lai, K.; Gu, Z. Effect of Sodium Oleate as a Buffer on the Synthesis of Superparamagnetic Magnetite Colloids. *J. Colloid Interface Sci.* **2010**, *347* (1), 1–7. https://doi.org/10.1016/j.jcis.2010.02.055.

(2) Sun, S.; Zeng, H.; Robinson, D. B.; Raoux, S.; Rice, P. M.; Wang, S. X.; Li, G. Monodisperse MFe 2 O 4 (M = Fe, Co, Mn) Nanoparticles. *J. Am. Chem. Soc.* **2004**, *126* (1), 273–279. https://doi.org/10.1021/ja0380852.

(3) Kim, B. H.; Lee, N.; Kim, H.; An, K.; Park, Y. Il; Choi, Y.; Shin, K.; Lee, Y.; Kwon, S. G.; Na, H. Bin; et al. Large-Scale Synthesis of Uniform and Extremely Small-Sized Iron Oxide Nanoparticles for High-Resolution T 1 Magnetic Resonance Imaging Contrast Agents. *J. Am. Chem. Soc.* **2011**, *133* (32), 12624–12631. https://doi.org/10.1021/ja203340u.

(4) Galli, M.; Guerrini, A.; Cauteruccio, S.; Thakare, P.; Dova, D.; Orsini, F.; Arosio, P.; Carrara, C.; Sangregorio, C.; Lascialfari, A.; et al. Superparamagnetic Iron Oxide Nanoparticles Functionalized by Peptide Nucleic Acids. *RSC Adv.* **2017**, *7* (25), 15500–15512. https://doi.org/10.1039/C7RA00519A.

(5) Hao, R.; Yu, J.; Ge, Z.; Zhao, L.; Sheng, F.; Xu, L.; Li, G.; Hou, Y. Developing Fe3O4 Nanoparticles into an Efficient Multimodality Imaging and Therapeutic Probe. *Nanoscale* **2013**, *5* (23), 11954–11963. https://doi.org/10.1039/c3nr04157c.

(6) Galli, M.; Rossotti, B.; Arosio, P.; Ferretti, A. M.; Panigati, M.; Ranucci, E.; Ferruti, P.; Salvati, A.; Maggioni, D. A New Catechol-Functionalized Polyamidoamine as an Effective SPION Stabilizer. *Colloids Surfaces B Biointerfaces* **2019**, *174* (October 2018), 260–269. https://doi.org/10.1016/j.colsurfb.2018.11.007.
